# Between-Batch Bioequivalence (BBE): a Statistical Test to Evaluate *In Vitro* Bioequivalence Considering the Between-Batch Variability

**DOI:** 10.1208/s12248-020-00486-5

**Published:** 2020-09-10

**Authors:** Jonathan Bodin, Stéphanie Liandrat, Gabriel Kocevar, Céline Petitcolas

**Affiliations:** 1 Data Science Department, Seenovate, Lyon, France; 2 Nemera, Insight Innovation Center, La Verpilliere, 20 avenue de la gare, 38290, La Verpilliere, France

**Keywords:** between-batch variability, equivalence test, in vitro bioequivalence, nasal spray, statistical test

## Abstract

Bioequivalence testing is an essential step during the development of generic drugs. Regulatory agencies have drafted recommendations and guidelines to frame this step but without finding any consensus. Different methodologies are applied depending on the geographical region. For instance, in the EU, EMA recommends using average bioequivalence test (ABE), while in the USA, FDA recommends using population bioequivalence (PBE) test. Both methods present some limitations (*e.g.*, when batch variability is non-negligible) making it difficult to conclude to equivalence without subsequently increasing the sample size. This article proposes an alternative method to evaluate bioequivalence: between-batch bioequivalence (BBE). It is based on the comparison between the mean difference (Reference − Test) and the Reference between-batch variability. After presenting the theoretical concepts, BBE relevance is evaluated through simulation and real case (nasal spray) studies. Simulation results showed high performance of the method based on false positive and false negative rate estimations (type I and type II errors respectively). Especially, BBE has shown significantly greater true positive rates than ABE and PBE when the Reference residual standard deviation is higher than 15%, depending on the between-batch variability and the number of batches. Finally, real case applications revealed that BBE is more efficient than ABE and PBE to demonstrate equivalence, in some well-known situations where the between-batch variability is not negligible. These results suggest that BBE could be considered as an alternative to the state-of-the-art methods allowing costless development.

**Electronic supplementary material:**

The online version of this article (10.1208/s12248-020-00486-5) contains supplementary material, which is available to authorized users.

**Figure:**
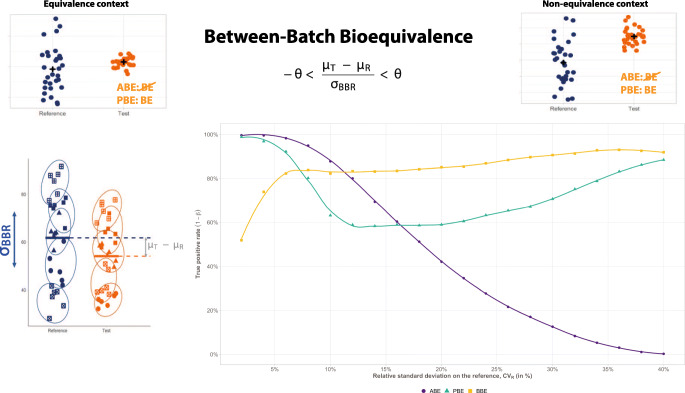
Graphical abstract

## INTRODUCTION

In the pharmaceutical industry, generics are becoming more and more important, mainly driven by countries’ regulations for giving patients easier access to drugs. From an industrial point of view, the final goal is to have a generic product that complies with all the regulatory requirements and is safe for the patient, *i.e.*, has the same final therapeutic effect than the brand-name product. One way to prove the equivalence, without long and expensive clinical trials, is to conduct *in vitro* bioequivalence evaluation between the Innovator (Reference product) and the proposed generic (Test product). The demonstration of equivalence could be conducted through statistical methods (1–6). *In vitro* bioequivalence testing is not considered and evaluated in the same way by all instances taking part in the process (7,8). Indeed, in the USA, FDA recommends the use of population bioequivalence (PBE) (9,10), whereas Europe recommends using average bioequivalence (ABE) (11,12).

On one side, ABE (12) is based on the two one-sided *t* test (TOST) developed by Schuirmann in 1987 (13). The method consists of comparing the difference between the Reference and Test means (arithmetic or geometric) to a preset equivalence limit *θ*_ABE_, by computing the 90% confidence interval of the mean difference. A log transformation may be applied prior to the ABE application depending on the data distribution (*e.g.*, with AUC). In opposition, PBE (2,14–19) systematically applies a log transformation to the data and scales the difference between the two geometric means according to the variability on the Reference product. Moreover, PBE induces an asymmetrical formula through the computation of the variance difference ${\sigma}_T^2-{\sigma}_R^2$. Therefore, PBE is more restrictive than ABE if the Reference product variability is low. Conversely, PBE is less restrictive than ABE if the Reference variability is high, even more so if the Test product variability is lower than that of the Reference product (11).

These statistical methods are different, and consequently provide different conclusions (1). Consequently, being bioequivalent using the different existing statistical methods on one device could be impossible. Recently, the European Federation for Pharmaceutical Sciences initiated work on a harmonization process to evaluate bioequivalence across the world with the aim of obtaining a robust and common approach (1). In addition, a recent report highlighted the difficulties on the statistic relating to *in vitro* or *in vivo* bioequivalence testing (2). This report highlights on the necessity to develop “more consistent, better aligned, science-based approaches” across countries (20).

ABE has a bioequivalence limit of 15%. The guidance recommends the calculation of 90% confidence intervals for the observed *in vitro* differences (2,21,22). Contrary to the FDA (14), EMA does not account for the parameter specificities and, particularly, the variability. This aspect, also called the one-size-fits-all criterion, may be a major concern for the bioequivalence test (23). Indeed, the confidence interval inflation, which depends on the variability and heteroscedasticity, may lead to reject the bioequivalence even when both products appear instinctively equivalent (5).

In the last decade, PBE has been challenged by several studies (1,2,9,21), especially in the context of *in vitro* bioequivalence testing. The main limitation is the asymmetry of the method, promoting situations with higher variability on the Reference product than on the Test product (9,21). In addition, the bioequivalence limit is fixed by the guidance on what may be questionable (21,24). Finally, as highlighted in Morgan *et al.* (2018), the IPAC-RS PBE working group recommends further investigation into the appropriateness of a log transformation for *in vitro* data in equivalence assessments, (9,21). Therefore, the asymmetrical formula of PBE may lead to accept the bioequivalence, even when both products appear instinctively not equivalent. This may increase the type I error, which reflects the probability to incorrectly accept equivalence. This situation appears especially when the Reference variability and heteroscedasticity are important (11).

In order to prevent the risks of erroneously accepting equivalence in cases of high Reference variability, it is recommended to consider more samples (4,5,25,26). This approach also allows to improve the power test (*i.e.*, the true positive rate TPR) on PBE and ABE calculations. However, even if this solution could be acceptable for *in vitro* studies, it could be ethically questionable for *in vivo* studies in order to limit exposures to patients.

While both ABE and PBE tests consider the device total variability, the between-batch variability, which is part of the last, is not considered individually in the mathematical definitions proposed by the FDA and EMA guidelines. Recent works recommended to consider the between-batch variability in the bioequivalence tests (2,9,21,27,28). Indeed, both methods do not consider the between-batch variability. On one side, ABE only considers within-product variability. On the other side, the FDA guidance on Budesonide (29) recommends to decompose the variance **as** a composite of a super-batch variability (*i.e.*, after pooling all batches per product) and the within-individual variability to study the life-stages (begin, middle, and end-of-use) equivalence. Burmeister *et al.* (27) illustrated the inability of the state-of-art methods to prove the equivalence between two batches of the same Reference product, which are by definition equivalent. Furthermore, Burmeister *et al.* (28) showed an increased probability, around + 25%, to incorrectly conclude on equivalence (*i.e.*, the type I error or false positive rate, FPR) in the presence of between-batch variations. In addition, Morgan *et al.* (9) confirmed the increase of at least 15% of the probability to incorrectly conclude on equivalence (type I error) when neglecting between-batch variability in PBE studies. This same study also highlights an increase of the probability to incorrectly reject equivalence (*i.e.*, the type II error or false negative rate, FNR) close to 20% in high between-batch variability cases. Indeed, authors obtained a type II error of 6% with no between-batch variability and 24% with 50% of the variability attributed to the between-batch, when the relative standard deviation is of 10%.The same magnitude is observed with higher relative standard deviation on the Reference [9]. Thus, considering this between-batch variability in the statistical formula has a potential to improve the probability to correctly accept the equivalence.

From this statement, an alternative statistical test, named between-batch equivalence (BBE), is proposed to assess *in vitro* bioequivalence. This statistical approach is based on the comparison between the mean difference (Reference − Test) and the Reference between-batch variability. The main hypothesis is that considering the between-batch variability of the reference, the BBE test will be more appropriate to demonstrate equivalence in the case of variable drug products, without needing to increase the total required sample size. This statistical method can deal with normal scale data as well as after log transformation of the raw data. As a first step, this study focused on the development of the statistical method, including an exact procedure to implement the test statistic and a confidence interval approximation to graphically illustrate the test results. Then, the type II error of the BBE method, *i.e.*, the FNR, was estimated by simulations and compared with the two mainly recognized statistical methods (ABE and PBE). In a second step, the type I error of the BBE method, *i.e.*, the FPR, was estimated by simulations to ensure that the BBE type I error remains of the theoretical order of 5%. In a third step, the BBE type II error was assessed and compared with the reference methods through a real case application on nasal spray *in vitro* performance that were performed on two Reference products from the market (which are by definition equivalent). Finally, the result interpretation and conclusions are drawn.

## MATERIAL AND METHODS

### Theory: The Between-Batch Bioequivalence Procedure

#### Statistical Model

As reported in several studies, neglecting non-zero between-batch variability can have a strong impact on the bioequivalence conclusions (21,27). Thus, the method developed in this work proposes to include the Batch factor into the statistical equivalence test. In fact, the multiple measurements on a single batch induce a dependence between data and a violation of the independence assumption with the state-of-the-art methods ABE and PBE. The assumption of independence could be satisfied if the Batch factor is considered in the model.

This BBE statistical context is close to the nested mixed model with a fixed effect Product, and a random effect Batch nested in Product.

$$ Y\sim Product+ Batch\left[ Product\right] $$

For such models, the significance test for the Product fixed effect is performed through the mean square (*MS*) ratio *MS*(*Product*)/*MS*(*Batch*[*Product*]), which follows a *F* distribution under the null hypothesis. Conceptually, the nested mixed model computes the ratio of the difference between the two means (Reference and Test) on one side and the mean difference between batches per product on the other side.

The mixed-effect model described above is a difference test, *i.e.*, the alternative hypothesis assumes the difference between the two products. In the context of equivalence testing, the alternative hypothesis assumes no difference between the two products. Furthermore, the mean square *Batch*[*Product*] considers the batches of the two products. Conceptually, the equivalence test should compare the difference between the two products with the differences between Reference batches. BBE can be formulated through the two following hypotheses:

$$ {H}_0:\frac{\mu_{\mathrm{T}}-{\mu}_{\mathrm{R}}}{\sigma_{\mathrm{BBR}}}\ge \theta\ \mathrm{or}\kern0.5em \frac{\mu_{\mathrm{T}}-{\mu}_{\mathrm{R}}}{\sigma_{\mathrm{BBR}}}\le -\theta $$

$$ {H}_1:-\theta <\frac{\mu_{\mathrm{T}}-{\mu}_{\mathrm{R}}}{\sigma_{\mathrm{BBR}}}<\theta \kern0.75em \left(\mathrm{Equivalence}\ \mathrm{between}\ \mathrm{products}\right), $$

where *μ*_T_ and *μ*_R_ stand for the means of the Test and Reference products measurements, *σ*_BBR_ for the between-batch variability on the Reference product, and *θ* for the bioequivalence limit. Thus, the method is based on the comparison between the mean difference (Reference − Test) and the Reference between-batch variability.

The proposed approach has been patented under the reference WO2020/053223 A1 (30).

#### Exact Procedure

Let us suppose throughout this section that the observation of each batch follows a normal distribution with a part of common variance. More specifically, ${\sigma}_{\mathrm{BBR}}^2={\sigma}_{\mathrm{BBT}}^2$, where ${\sigma}_{\mathrm{BBT}}^2$ stands for the between-batches variance of the Test products.

Under this assumption, the batch means samples of the Reference and Test, respectively ${\overline{x}}_{{\mathrm{B}}_i\mathrm{R}}$ and ${\overline{x}}_{{\mathrm{B}}_i\mathrm{T}}$, follow a Gaussian distribution ${\overline{x}}_{{\mathrm{B}}_i\mathrm{R}}\sim \mathcal{N}\left({\mu}_{\mathrm{R}},{\sigma}_{\mathrm{B}\mathrm{BR}}^2\right)$ and ${\overline{x}}_{{\mathrm{B}}_i\mathrm{T}}\sim \mathcal{N}\left({\mu}_{\mathrm{T}},{\sigma}_{\mathrm{B}\mathrm{BR}}^2\right)$.

From the *H*_0_/*H*_1_ hypotheses testing, BBE can be endorsed if the probability of −*θ* < *δ* < *θ* is greater than the confidence level (1 − *α*), with *δ* = (*μ*_T_ − *μ*_R_)/*σ*_BBR_. An estimator of *δ* can be expressed as $g=\left({\overline{x}}_{\mathrm{T}}-{\overline{x}}_{\mathrm{R}}\right)/{s}_{\mathrm{BBR}}$. However, the sampling distribution of *g* cannot be analytically determined. Based on (27), the sampling distribution of $\frac{g}{K}$ can be calculated if *K* is a constant, defined by:

$$ K=\sqrt{\frac{n_{\mathrm{BT}}+{n}_{\mathrm{BR}}}{n_{\mathrm{BT}}{n}_{\mathrm{BR}}}}, $$

where ${n}_{\mathsf{BR}}$ denotes the number of sampled batches for the Reference product and ${n}_{\mathsf{BT}}$ is the number of sampled batches for the Test product.

Then, $\frac{g}{K}=\frac{{\overline{x}}_{\mathrm{T}}-{\overline{x}}_{\mathrm{R}}}{s_{\mathrm{BBR}}}\times \sqrt{\frac{n_{\mathrm{BT}}\ {n}_{\mathrm{BR}}}{n_{\mathrm{BT}}+{n}_{\mathrm{BR}}}}$ follows a non-centered Student distribution (${\mathcal{T}}_{\mathrm{nc}}$) with *n*_BR_ − 1 degrees of freedom and a noncentrality parameter equal to

$$ \frac{\mu_{\mathrm{T}}-{\mu}_{\mathrm{R}}}{\sigma_{\mathrm{BBR}}}\times \sqrt{\frac{n_{\mathrm{BT}}\ {n}_{\mathrm{BR}}}{n_{\mathrm{BT}}+{n}_{\mathrm{BR}}}}. $$

Furthermore, Hedges (31) demonstrated that $g=\left({\overline{x}}_{\mathrm{T}}-{\overline{x}}_{\mathrm{R}}\right)/{s}_{\mathrm{BBR}}$ is a biased estimator of *δ*. An unbiased estimator of *δ* can be obtained through the application of a correction function, *c*,that only depends on the degrees of freedom of *s*_R_. This correction function can be approximated by

$$ c\left({n}_{BR}\right)\approx 1-\frac{3}{4\left({n}_{BR}-1\right)-1}. $$

We can then infer that an unbiased estimator of *δ* = (*μ*_T_ − *μ*_R_)/*σ*_BBR_ is given by

$$ \frac{{\overline{x}}_{\mathrm{T}}-{\overline{x}}_{\mathrm{R}}}{s_{\mathrm{BBR}}}\times \left[1-\frac{3}{4\left({n}_{\mathrm{BR}}-1\right)-1}\right]. $$

Thus, the BBE test statistic follows a noncentral Student distribution with *n*_BR_ − 1 degrees of freedom.

The BBE test statistic $\left({\overline{x}}_{\mathrm{T}}-{\overline{x}}_{\mathrm{R}}\right)/{s}_{\mathrm{BBR}}$is distributed as

$$ \sqrt{\frac{\ {n}_{\mathrm{BT}}+{n}_{\mathrm{BR}}}{n_{\mathrm{BT}}\ {n}_{\mathrm{BR}}}}\times \frac{1}{c\left({n}_{\mathrm{BR}}-1\right)}\times \mathcal{T}, $$

where $\mathcal{T}$ is a noncentral Student law with ${n}_{\mathsf{BR}}-1$ degrees of freedom and noncentrality parameter

$$ \frac{\mu_{\mathrm{T}}-{\mu}_{\mathrm{R}}}{\sigma_{\mathrm{BBR}}}\times \sqrt{\frac{n_{\mathrm{BT}}\ {n}_{\mathrm{BR}}}{n_{\mathrm{BT}}+{n}_{\mathrm{BR}}}}. $$

The *H*_1_ alternative hypothesis can be written as

$$ {H}_1:-\theta \times \sqrt{\frac{n_{\mathrm{BT}}\ {n}_{\mathrm{BR}}}{n_{\mathrm{BT}}+{n}_{\mathrm{BR}}}}<\frac{\mu_{\mathrm{T}}-{\mu}_{\mathrm{R}}}{\sigma_{\mathrm{BBR}}}\times \sqrt{\frac{n_{\mathrm{BT}}\ {n}_{\mathrm{BR}}}{n_{\mathrm{BT}}+{n}_{\mathrm{BR}}}}<\theta \times \sqrt{\frac{n_{\mathrm{BT}}\ {n}_{\mathrm{BR}}}{n_{\mathrm{BT}}+{n}_{\mathrm{BR}}}}. $$

Thus, the method of the BBE test consists of calculating the test statistic

$$ {T}_{\mathrm{BBE}}=\frac{{\overline{x}}_{\mathrm{T}}-{\overline{x}}_{\mathrm{R}}}{s_{\mathrm{BBR}}}\times \sqrt{\frac{n_{\mathrm{BT}}\ {n}_{\mathrm{BR}}}{n_{\mathrm{BT}}+{n}_{\mathrm{BR}}}}\times \left[1-\frac{3}{4\left({n}_{\mathrm{BR}}-1\right)-1}\right] $$

In conclusion, if

$$ {T}_{\mathrm{BBE}}<{\mathcal{T}}_{\mathrm{nc}}\left(0.05;{n}_{\mathrm{BR}}-1;\theta \times \sqrt{\frac{n_{\mathrm{BT}}\ {n}_{\mathrm{BR}}}{n_{\mathrm{BT}}+{n}_{\mathrm{BR}}}}\right) $$

and

$$ {T}_{\mathrm{BBE}}>{\mathcal{T}}_{\mathrm{nc}}\left(0.95;{n}_{\mathrm{BR}}-1;-\theta \times \sqrt{\frac{n_{\mathrm{BT}}\ {n}_{\mathrm{BR}}}{n_{\mathrm{BT}}+{n}_{\mathrm{BR}}}}\right), $$

then, the *H*_1_ hypothesis (*i.e.*, the equivalence between the two products) is endorsed with ${\mathcal{T}}_{\mathsf{nc}}$ the noncentral Student distribution quantile.

#### Bioequivalence Limits

Conceptually, the Test can be considered statistically equivalent to the Reference if its mean is comprised inside the 95% tolerance interval of the Reference batch means. Based on the central limit theorem, it can be inferred that the means of the Reference batches follow a normal distribution of mean *μ*_R_ and variance ${\sigma}_{\mathrm{BBR}}^2$: ${\overline{x}}_{{\mathrm{B}}_i\mathrm{R}}\to \mathcal{N}\left({\mu}_{\mathrm{R}},{\sigma}_{\mathrm{B}\mathrm{BR}}\right)$. Thus, 95% of the means of the Reference product batches are included in the interval [*μ*_R_ − 1.96 *σ*_BBR_; *μ*_R_ + 1.96 *σ*_BBR_].

Therefore, in the remaining parts of this work, the Test will be considered statistically equivalent to the Reference if

$$ -\theta <\frac{\mu_{\mathrm{T}}-{\mu}_{\mathrm{R}}}{\sigma_{\mathrm{BBR}}}<\theta, \kern0.75em \mathrm{with}\kern0.5em \theta =1.96 $$

BBE bioequivalence limit will be challenged on TPR estimation through different real cases.

#### BBE Computation

1. Estimate the BBE test statistic
  $$ {T}_{\mathrm{BBE}}=\frac{{\overline{x}}_T-{\overline{x}}_R}{s_{\mathrm{BBR}}}\sqrt{\frac{c\left({n}_{\mathrm{BR}}\right)}{K}},\kern0.5em \mathrm{with}\kern0.75em c\left({n}_{BR}\right)\approx 1-\frac{3}{4\left({n}_{BR}-1\right)-1}\kern0.75em \mathrm{and}\kern0.5em K=\sqrt{\frac{n_{\mathrm{BR}}+{n}_{\mathrm{BT}}}{n_{\mathrm{BR}}\ {n}_{\mathrm{BT}}}} $$

##### Estimate the bioequivalence limits

$$ {\displaystyle \begin{array}{c}\mathrm{Li}{\mathrm{m}}_{\mathrm{Inf}}={\mathcal{T}}_{\mathrm{nc}}\left(0.95;{n}_{\mathrm{BR}}-1;-\frac{\theta }{K}\right),\\ {}\mathrm{Li}{\mathrm{m}}_{\mathrm{Sup}}={\mathcal{T}}_{\mathrm{nc}}\left(0.05;{n}_{\mathrm{BR}}-1;\frac{\theta }{K}\right).\end{array}} $$

When *n*_BT_ = *n*_BR_, the bioequivalence limits are the following

- 3 batches: ]−0.7395, 0.7395[
- 4 batches: ]−1.0666, 1.0666[
- 5 batches: ]−1.3581, 1.3581[
- 6 batches: ]−1.6247, 1.6247[
- 7 batches: ]−1.8722, 1.8722[
- 8 batches: ]−2.1044, 2.1044[
- 9 batches: ]−2.3239, 2.3239[
- 10 batches: ]−2.5328, 2.5328[

In order to estimate the noncentral Student distribution when *n*_BT_ ≠ *n*_BR_, one can use statistical software or online calculator, such as (32).

*BBE Is Endorsed if Lim*_*Inf*_ < *T*_*BBE*_ < *Lim*_*Sup*_

#### Graphical illustration of BBE results: BBE_CI_

In order to illustrate the BBE results, a confidence interval of the BBE test statistic is proposed: BBE_CI_. Similarly to the approach developed for the PBE method (7), the moment method is applied.

First, let us linearize the test hypotheses *H*_0_/*H*_1_,

$$ {\displaystyle \begin{array}{c}{H}_0:\kern0.5em \frac{{\left({\mu}_{\mathrm{T}}-{\mu}_{\mathrm{R}}\right)}^2}{\sigma_{\mathrm{BBR}}^2}\ge {\theta}^2\Longleftrightarrow {H}_0:{\left({\mu}_{\mathrm{T}}-{\mu}_{\mathrm{R}}\right)}^2-{\theta}^2\times {\sigma}_{\mathrm{BBR}}^2\ge 0\\ {}{H}_1:\kern0.5em \frac{{\left({\mu}_{\mathrm{T}}-{\mu}_{\mathrm{R}}\right)}^2}{\sigma_{\mathrm{BBR}}^2}<{\theta}^2\Longleftrightarrow {H}_1:{\left({\mu}_{\mathrm{T}}-{\mu}_{\mathrm{R}}\right)}^2-{\theta}^2\kern0.5em \times {\sigma}_{\mathrm{BBR}}^2<0\end{array}} $$

The punctual estimation of the BBE_CI_ test is then given by:

$$ {E}_{\lambda }={\left({\overline{x}}_{\mathrm{T}}-{\overline{x}}_{\mathrm{R}}\right)}^2-{\theta}^2\times {s}_{\mathrm{BBR}}^2 $$

The 95% confidence interval upper limit of *E*_*λ*_ must be strictly negative to accept the alternative hypothesis. This upper limit is given by the following coefficient noted *H*_*λ*_:

$$ {H}_{\lambda }={E}_{\lambda }+\sqrt{{\left(\mathrm{Estimation}\ \mathrm{error}\ \mathrm{of}\ {\left({\mu}_{\mathrm{T}}-{\mu}_{\mathrm{R}}\right)}^2\right)}^2+{\left(\mathrm{Estimation}\ \mathrm{error}\ \mathrm{of}\ \left(-{\theta}^2\times {\sigma}_{\mathrm{BBR}}^2\right)\right)}^2} $$

Thus, the 95% confidence interval upper limit of the BBE_CI_ test is given by:

$$ {H}_{\lambda }={\left({\overline{x}}_{\mathrm{T}}-{\overline{x}}_{\mathrm{R}}\right)}^2-{\theta}^2\times {s}_{\mathrm{BBR}}^2+\sqrt{\begin{array}{c}{\left({\left(\left|{\overline{x}}_{\mathrm{T}}-{\overline{x}}_{\mathrm{R}}\right|+{t}_{1-\alpha, \kern0.5em \mathrm{df}}\sqrt{\frac{s_{\mathrm{T}}^2}{n_{\mathrm{T}}}+\frac{s_{\mathrm{R}}^2}{n_{\mathrm{R}}}}\right)}^2-{\left({\overline{x}}_{\mathrm{T}}-{\overline{x}}_{\mathrm{R}}\right)}^2\right)}^2+\dots \\ {}\ {\left(-{\theta}^2\times \frac{\left({n}_{\mathrm{BR}}-1\right)\ {s}_{\mathrm{BBR}}^2}{\chi_{1-\alpha, \kern0.75em {n}_{\mathrm{BR}}-1}^2}+{\theta}^2\times {s}_{\mathrm{BBR}}^2\right)}^2\end{array}} $$

where $\mathsf{df}$ is the degree of freedom of the student coefficient after Welch-Satterthwaite correction

$$ \mathrm{df}\left(\mathrm{Welch}-\mathrm{Satterthwaite}\right)=\frac{{\left({SEM}_{\mathrm{T}}^2+{SEM}_{\mathrm{R}}^2\right)}^2}{\frac{SEM_{\mathrm{T}}^4}{n_{\mathrm{T}}-1}+\frac{SEM_{\mathrm{R}}^4}{n_{\mathrm{R}}-1}}=\frac{{\left(\frac{s_{\mathrm{T}}^2}{n_{\mathrm{T}}}+\frac{s_{\mathrm{R}}^2}{n_{\mathrm{R}}}\right)}^2}{\frac{s_{\mathrm{T}}^4}{n_{\mathrm{T}}^2\left({n}_{\mathrm{T}}-1\right)}+\frac{s_{\mathrm{R}}^4}{n_{\mathrm{R}}^2\left({n}_{\mathrm{R}}-1\right)}} $$

### Experimental: Simulation Design

Simulation studies were processed to estimate and assess the proposed method type I and type II errors, *i.e.*, the false positive and false negative rates respectively. The type II error simulations were computed for the three equivalence methods (ABE, PBE, and BBE) in order to compare their ability to accurately prove equivalence. Simulations consisted of 10,000 replications for each combination of triplets (*σ*_R_, *π*_BB_, *n*_Batches_). As detailed in Table I, a wide range of different input parameters were used to generate the simulated dataset. These parameters, including the number of batches ranging from 3 to 10, RSD ranging from 5% to 40%, and the part of variability attributed to between-batch variability ranging from 10% to 90% were used to fit a large variety of real cases.

**Table I:** Input Parameters Provided to the Simulation Algorithm

Parameter	Notation	Value(s)	Different values
Initial sample size	*n* = *n*_R_ = *n*_T_	30	1
Number of batches per product	*n*_Batches_	[3,4,5,6,7,8,9,10]	8
Reference mean	*μ*_R_	10	1
Test mean	*μ*_T_	*μ*_T_ = *μ*_R_ = 10 (Type II error)*μ*_T_ = *μ*_R_ + 1.96 × *σ*_BBR_ (Type I error) *μ*_T_ = *μ*_R_ + (1.96 × *σ*_BBR_) × 1.05 (Type I error) *μ*_T_ = *μ*_R_ + (1.96 × *σ*_BBR_) × 1.1 (Type I error)	4
Standard deviation	*σ* = *σ*_R_ = *σ*_*T*_	[0.5, 0.6, 0.7, 0.8, 0.9, 1, 1.1, 1.2, 1.3, 1.4, 1.5, 1.6, 1.7, 1.8, 1.9, 2, 2.1, 2.2, 2.3, 2.4, 2.5, 2.6, 2.7, 2.8, 2.9, 3, 3.1, 3.2, 3.3, 3.4, 3.5, 3.6, 3.7, 3.8, 3.9, 4]	36
Between-batch variability/total variability	*π*_BB_ =*π*_BBR_ =*π*_BBT_	[0.1, 0.2, 0.3, 0.4, 0.5, 0.6, 0.7, 0.8, 0.9]	9

Four different mean values of the Test population were used:

- The first one, *μ*_T_ = *μ*_R_, was used to estimate the true positive rate (TPR) *i.e.*, the power test (1 − type II error). The results are analyzed and compared with the three equivalence methods (ABE, PBE, and BBE) with respect to the triplet [*σ*_R_, *π*_BB_, *n*_Batches_].
- The three last ones were computed to estimate the false positive rate (FPR), *i.e.*, the type I error. The results are analyzed for the BBE method with respect to the same triplet.
- *μ*_T_ = *μ*_R_ + 1.96 *σ*_BBR_ corresponding to BBE equivalence limit
- *μ*_T_ = *μ*_R_ + 1.96*σ*_BBR_ × 1.05 representing a deviation of 5% from the bioequivalence limit.
- *μ*_T_ = *μ*_R_ + 1.96*σ*_BBR_ × 1.1 representing a deviation of 10% from the bioequivalence limit.

The number of products (*n*_PB_) composing each batch is defined as the round value of the ratio *n*/*n*_Batches_. Then, *n* = 32 for 4 and 8 batches; *n* = 30 for 3, 5, 6, and 10 batches; *n* = 28 for 7 batches; and *n* = 27 for 9 batches.

To compute the between-batch (*σ*_BBR_) and the within-batch (*σ*_WBR_) variabilities, the sum of squares had to be calculated:

- The total sum of squares $S{S}_{\mathrm{Tot}}={\sigma}_{\mathrm{R}}^2\times \left({n}_{\mathrm{R}}-1\right)$,
- The between-batch sum of squares *SS*_BB_ = *π*_BB_ × *SS*_Tot_,
- The within-batch sum of squares *SS*_WB_ = *SS*_Tot_ × (1 − *π*_BB_),

where *π*_BB_ denotes the percentage of the total variability attributed to the between-batch variability. Then,

$$ {\displaystyle \begin{array}{c}{\sigma}_{\mathrm{BB}\mathrm{R}}=\sqrt{\frac{S{S}_{\mathrm{BB}}}{n_{\mathrm{PB}}\times \left({n}_{\mathrm{Batches}}-1\right)}}=\sqrt{\frac{\pi_{\mathrm{BB}}\times {\sigma}_{\mathrm{R}}^2\times \left({n}_{\mathrm{R}}-1\right)}{n_{\mathrm{PB}}\times \left({n}_{\mathrm{Batches}}-1\right)}}\ \mathrm{and}\\ {}{\sigma}_{\mathrm{WB}\mathrm{R}}=\sqrt{\frac{S{S}_{\mathrm{WB}}}{n_{\mathrm{R}}-{n}_{\mathrm{Batches}}}}=\sqrt{\frac{\sigma_{\mathrm{R}}^2\times \left({n}_{\mathrm{R}}-1\right)\times \left(1-{\pi}_{\mathrm{BB}}\right)}{n_{\mathrm{R}}-{n}_{\mathrm{Batches}}}}.\end{array}} $$

The following procedure was computed to simulate the Reference data (the same approach is applied to simulate the Test product).

1. *Estimation of batch means values* (${\overline{x}}_{{\mathrm{B}}_i\mathrm{R}}$)

simulation of *n*_Batches_ data from a normal distribution ${\overline{x}}_{{\mathrm{B}}_i\mathrm{R}}\to \mathcal{N}\left({\mu}_{\mathrm{R}},{\sigma}_{\mathrm{B}\mathrm{BR}}\right)$

2. *Estimation of the j products parameter values inside each batch*
  - Estimation of a normal distribution with null mean and standard deviation of *σ*_WBR_: ${x}_{{\mathrm{B}}_{ij}\mathrm{R}0}\to \mathcal{N}\left(0,{\sigma}_{\mathrm{WBR}}\right)$. Due to simulations, the real mean of this distribution is not exactly equal to 0. Then, this distribution is centered back to a mean of 0 by subtracting to ${x}_{{\mathrm{B}}_{ij}\mathrm{R}0}$ the mean of ${x}_{{\mathrm{B}}_{ij}\mathrm{R}0}$: ${x}_{{\mathrm{B}}_{ij}\mathrm{R}0}={x}_{{\mathrm{B}}_{ij}\mathrm{R}0}-\frac{1}{n_{\mathrm{B}}}{\sum}_{j=1}^{n_{\mathrm{B}}}{x}_{{\mathrm{B}}_{ij}\mathrm{R}0}$
  - Finally, for each product (*j*) parameter value (${x}_{{\mathrm{B}}_{ij}\mathrm{R}}$) of each batch (*i*) is estimated by summing ${x}_{{\mathrm{B}}_{ij}\mathrm{R}0}$ to its corresponding batch mean value: ${x}_{{\mathrm{B}}_{ij}\mathrm{R}}={x}_{{\mathrm{B}}_{ij}\mathrm{R}0}+{\overline{x}}_{{\mathrm{B}}_i\mathrm{R}}$.

ABE (*T*_ABE_), PBE (*T*_PBE_), and BBE (*T*_BBE_) test statistics were estimated for the 10,000 replications of each triplet [*σ*_R_, *π*_BB_, *n*_Batches_] and the TPR of each method was calculated. As required, a log transformation was applied to the data before estimating the PBE test statistic. No data transformation was applied to perform ABE and BBE. An equivalent procedure was applied to estimate the BBE FPR. Simulations and statistical analysis were computed using R version 3.6.1 (31).

### Experimental: Real Cases

The BBE approach has been applied to two nasal sprays, Nasonex® (Merck) and Flonase® (GSK), which are corticosteroids used to treat nasal symptoms such as congestion, sneezing and runny nose caused by seasonal or year-round allergies. These two products are already commercialized and considered individually as Reference products. In accordance with FDA guidance (16,19,33), two main parameters of *in vitro* spray performance were considered for the bioequivalence tests. The control of theses parameters ensures the quality of the nasal spray product and at the end the efficacy of patient treatment. The first, Dv50 (or D50) related to droplet size distribution (DSD), (34) was measured by laser diffraction using a Spraytec (Malvern, UK) and an automatic actuator (Proveris, USA). Second, the spray pattern (area) was measured using a Sprayview system (Proveris, USA). This parameter, describing the shape of the spray (35) is known for its high variability (36). The study has been performed at two distances (3 cm and 6 cm) as recommended in the guidance (18) with an actuation speed of 80 mm/s. Only data for Dv50 at a distance of 3 cm and a spray pattern area at a distance of 6 cm are presented here.

The statistical comparison of the three methods (ABE, PBE, and BBE) was performed separately on batches of Flonase and Nasonex products. After checking for the Normality of the distribution, the raw data were not transformed for ABE and BBE, except for the PBE which requires a log transformation. The aim was to evaluate whether the three methods are able to adequately conclude in an equivalence context of each product. For that purpose, the true positive rate of each method was estimated and compared. For both criteria (D50 and area) and both products (Nasonex and Flonase), batches of each product were randomly selected to be considered as a Reference or Test for the need of the bioequivalence evaluation (26).

Twenty-three batches of Flonase, each composed of 10 products, and 16 batches of Nasonex, each composed of 6 products, were used. For the Flonase product, all the possible combinations (without repetition and without replacement) of twice 3 batches (3 considered as the Reference and 3 considered as the Test) were computed. In the same way, all the possible combinations (without repetition and without replacement) of twice 5 batches of the Nasonex product (**5** considered as the Reference and **5** considered as the Test) were computed. For both the products, this resulted in comparing 30 products in the Reference and Test populations. A total of 2,018,940 combinations for the Flonase product and 2,018,016 combinations for the Nasonex were obtained and the 3 methods were applied to the D50 and area criteria.

As both Flonase and Nasonex products are commercial products, the selected batches can be considered as equivalent (all the Flonase batches are equivalent to all the other ones and all the Nasonex batches are equivalent to all the other ones). Thus, the ratio between the number of positive equivalence results to the total number of tests (*i.e.*, the total number of combinations) reflects the TPR.

## RESULTS

### Simulation Results: Type II Error

**Fig. 1:**
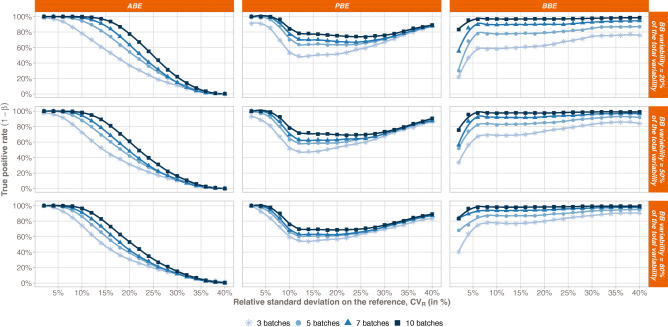
Bioequivalence true positive rates (power test profiles, *y*-axis) by method (ABE, PBE, and BBE in columns) with respect to the relative standard deviation on the Reference (CV_R_, *x*-axis). The comparison is done taking into consideration different numbers of batches (from 3 to 10 batches). Each line corresponds to different values of the between-batch variability (from 20 to 80%)

The true positive rate (TPR) comparison between the 3 statistical methods is compiled in Fig. 1 with respect to the Reference variability. From a general point of view, we can observe that the more batches in the Reference, the greater the TPR, whatever the bioequivalence method. Furthermore, each test exhibits a specific trend when looking at the relationship between the TPR and the total variability on the Reference.

For ABE, a decreasing sigmoid is observed, showing that the higher the Reference variability, the lower the ABE TPR. Moreover, an inflection point can be identified in terms of *CV*_R_. Before this point, the ABE TPR is close to 1, while after the ABE TPR decreases and tends to 0 when *CV*_R_ tends to infinity. This inflection point depends on both the number of batches in the Reference and the proportion of the total variability attributed to the between-batch variability.

For PBE, an inflection point can also be identified. Before this inflection point, the PBE TPR decreases from values close to 1 to its minimal value, while after, the PBE TPR slowly increases and tends to 1. However, contrary to ABE, this inflection point has a fixed *CV*_R_ value around 10%. It should be noted that the results only present situations where both the Reference and Test variabilities are equal. The simulations are performed with equal variances, *i.e.*, under homoscedasticity assumption.

An inflection point is also observed for BBE, corresponding to a *CV*_R_ value of 6%. This point is characterized by a sharp rise of TPR for *CV*_R_ values lower than 6%. TPR approaches the asymptote *y* = 1 when *CV*_R_ values are greater than 6%.

Going deeper in details, a plateau is observed for ABE, with TPR values close to 1, which length depends on both the number of batches in the Reference and the proportion of the total variability attributed to the between-batch variability (*π*_BB_). Indeed, the more batches in the Reference, the longer the plateau. In opposition, the higher the between-batch variability, the smaller the plateau. Thus, the ABE performance is the highest for small *CV*_R_ values, small *π*_BB_, and high number of batches.

The PBE true positive rate also depends on the number of batches in the Reference, with higher TPR values observed for the highest number of batches in the Reference. However, no strong relationship was observed between the TPR and the between-batch variability.

Concerning the BBE test, the TPR also depends on the number of batches in the Reference, with a TPR rise with the number of batches. The BBE TPR also depends on the between-batch variability, with a global increase of the TPR function of *π*_BB_. However, this dependence is less marked when the Reference number of batches increases. For instance, considering 3 batches and a total variability of 10%, the BBE TPR goes from 58% when *π*_BB_ = 20% to 84% when *π*_BB_ = 80%, while considering ten batches and a total variability of 10%, the BBE TPR goes from 97% when *π*_BB_ = 20% to 98% when *π*_BB_ = 80%.

**Fig. 2:**
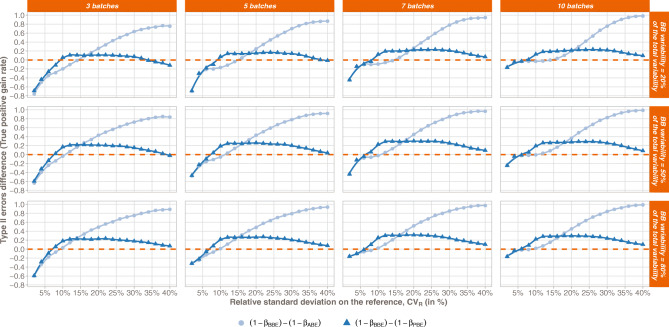
True positive rates comparison: BBE versus ABE (dark-blue curves) and BBE versus PBE (light-blue curves). Differences are expressed with respect to the number of batches (lines), the between-batch variability (π_BB_, rows), and the relative standard deviation on the Reference (RSD, x-axis). Positive values illustrate the higher power of BBE test in comparison with the respective TPR of ABE and PBE tests

The true positive rates (TPR) of the two state-of-the-art methods were compared with the BBE TPR and the differences between them are reported in Fig. 2. From a general point of view, results showed that the BBE method is more appropriate than the ABE and PBE methods for the high value of reference total variability.

More in detail, when compared with the ABE, the BBE method showed higher TPR from *CV*_R_ values around 15% when the between-batch variability equals 20% of the total variability, between 10% and 15% for 50% of the total variability attributed to the between-batch variability, and around 10% when the between-batch variability represents 80% of the total variability. Moreover, the higher the total variability, the higher the TPR gain.

When comparing BBE with PBE, the *CV*_R_ threshold to reach higher TPR is lower than the one obtained with ABE, while the TPR gain is lower. Indeed, this threshold is reached for *CV*_R_ values slightly varying between 5 and 10%, depending on the proportion of the total variability attributed to the between-batch variability. For high *CV*_R_ values, the TPR difference between PBE and BBE decreases and tends to 0. The maximal gain between PBE and BBE is around 30%, while this value grows to more than 90% when considering ABE versus BBE.

**Fig. 3:**
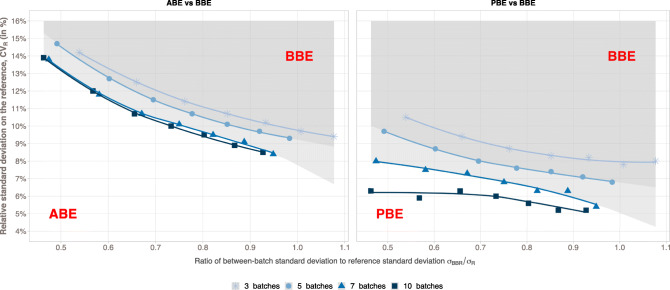
Performance of BBE method compared with ABE (left) and PBE (right) methods. Results, reported for 3, 5, 7, and 10 batches, show the interval, in terms of relative standard deviation on the Reference and of between-batch to the Reference standard deviations ratios, where BBE reach greater true positive rates than the two other methods (gray areas)

Thus, depending on the properties of the sampled batches, one method can reach higher TPR than the two others. Cutoff values have been identified and reported in Fig. 3. This figure delimits the area where the performance of one method is stronger than the others with respect to Reference variability (*y*-axis) and the part of the variability that is attributed to the between-batch (*x*-axis). The upper part (gray area) corresponds to the parameters for which BBE is stronger than ABE or PBE whereas the lower area corresponds to the opposite. For instance, with 5 batches, the BBE method reached higher power test values than ABE when the variability of the Reference is around 13% and the part of the variability explained by the between-batch is from 60%. If the variability on the Reference increases up to 15%, BBE is beneficial when the part of variability due to between-batch is around 50%. With a lower variability on the Reference (around 10%), BBE is stronger when the part of the Reference variability explained by the between-batch is around 85%. Taking the same example (**5** batches) to compare BBE and PBE, BBE reached higher TPR when the Reference variability is around 8% and the part of the variability explained by the between-batch around 70%. With 7 batches, it becomes advantageous to use BBE with a Reference variability around 8%, whatever the part of the variability explained by the between-batch variability.

**Fig. 4:**
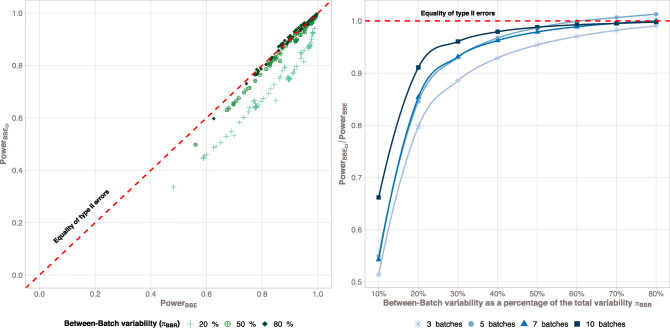
Comparison of BBE and BBE_CI_ method true positive rates (TPR). The left panel shows BBE_CI_ method TPR as a function of BBE TPR. Colors represent the between-batch variability (π_BBR_), expressed as a percentage of the total variability, arbitrarily discretized to the values of 20%, 50%, and 80%. The right panel shows BBE_CI_ to BBE power tests ratio with respect to the between-batch variability. Colors represent the number of batches

The relation between the graphical illustration of BBE (BBE Confidence Interval: BBE_CI_) and BBE is illustrated in Fig. 4. The graph on the left is the representation of BBE_CI_ TPR as a function of the BBE TPR. Results are colored upon different configurations of between-batch variability. This figure shows that a vast majority of the measurement are represented close to the line *y* = *x*, meaning that TPR of both tests are close. In addition, for a small number of observations, measurements slightly deviate from this line, with BBE_CI_ power test being always lower than BBE ones. On the right side, the graph represents the ratio between BBE_CI_ and BBE test with respect to the between-batch variability. When the between-batch variability increases, the ratio of BBE_CI_ to the BBE power tests increase and converges to 1, showing that BBE_CI_ is particularly relevant in non-zero between-batch variability situations (ratio greater than 0.9 when *π*_BB_ > 30%).

### Simulation Results: Estimation of the Relative Difference Between Means

With a similar simulation approach, BBE type II error was also estimated with respect to the relative difference between Reference and Test means. In that aim, the Reference mean was fixed at *μ*_R_ = 10 and the following values were used for the Test mean *μ*_T_ ∈ [10, 20], corresponding to the relative difference between means included in [0%, 100%].

**Fig. 5:**
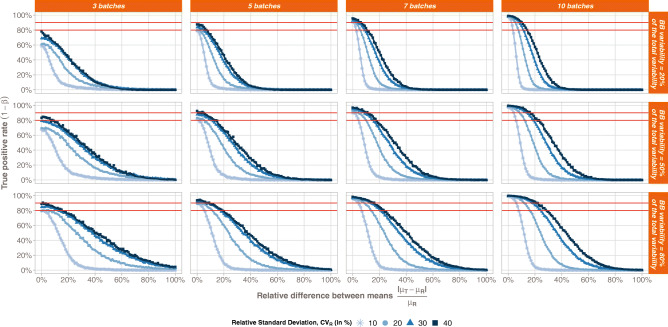
Bioequivalence true positive rates (power test profiles, *y*-axis) with respect to the relative difference between Reference and Test means. The comparison is done taking into consideration different number of batches (from 3 to 10 batches, in columns) and different Relative Standard Deviation values (*CV*_R_ = 10 % , 20 % , 30 % , 40%). Each line corresponds to different values of the between- batch variability (from 20% to 80%)

First, Fig. 5 confirmed the TPR values observed in Fig. 1 corresponding to the relative difference between means equal to 0. The main objective of Figure 5 is to illustrate the acceptable relative difference between means at fixed TPR levels in function of RSD, *π*_BB_ and *n*_BR_ values.

As expected, the acceptable relative difference increases with the RSD and the *π*_BB_, which together represent the Reference between-batch variability (*σ*_BBR_). Indeed, considering 5 batches on the Reference product and *CV*_R_ = 40%, the acceptable relative difference to reach a TPR greater than 80% is around 8% when the Reference between-batch variability representing 20% of the total variability (*π*_BB_ = 20%), around 15% with *π*_BB_ = 50%, around 20% with *π*_BB_ = 80%. On the other hand, considering 5 batches on the Reference product and *π*_BB_ = 50%, the acceptable relative difference to reach a TPR greater than 80% is around 2% when the Reference Relative Standard Deviation is equal to 10% (*CV*_R_ = 10%), around 8% with *CV*_R_ = 20%, around 12% with *CV*_R_ = 30%, around 14% with *CV*_R_ = 40%.

Similarly, the acceptable relative difference increases with the number of batches. For instance, with *π*_BB_ = 50%, *CV*_R_ = 40 % , and TPR = 80 % , the maximum difference between Reference and Test means is around 8% with 3 batches, around 15% with 5 batches, around 18% with 7 batches, and finally around 22% with 10 batches.

### Simulation Results: Type I Error

**Fig. 6:**
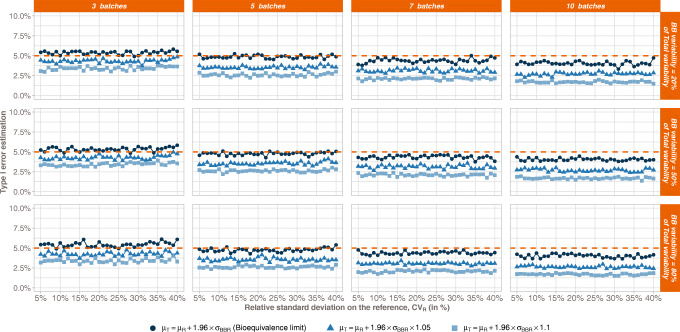
BBE type I error as a function of the relative standard deviation on the Reference. Type I errors are estimated for three values of the Test mean, namely μ_T_ = μ_R_ + 1.96σ_BBR_, corresponding to the bioequivalence limit, μ_T_ = μ_R_ + 1.96σ_BBR_ + ϵ, and μ_T_ = μ_R_ + 1.96σ_BBR_ + 2ϵ

Figure 6 illustrates the BBE false positive rate (type I error), as a function of the Reference variability. Observations are made upon the number of studied batches (columns, from 3 to 10 batches) and under consideration of the part of the Reference variability explained by the between-batch (lines, from 20 to 80%). The 3 curves represent different values of the Test mean. The first one corresponds to the BBE bioequivalence limit, the second and third ones to the bioequivalence limit plus a small deviation.

The first statement on this figure is that the BBE type I error for all configurations is equal to 5% or less. From the analysis of the figure, the type I error is independent of the part of the Reference variability that is linked to between batch whereas the type I error decreases when the number of studied batches increases.

**Fig. 7:**
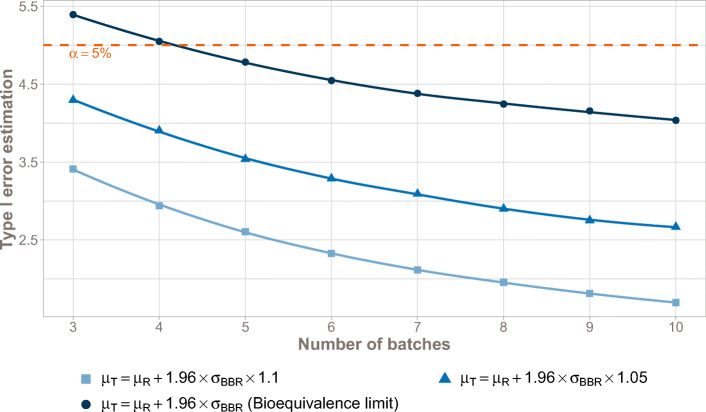
BBE type I error as a function of the number of batches. Type I errors are estimated for three values of the Reference mean, namely μ_T_ = μ_R_ + 1.96σ_BBR_, corresponding to the bioequivalence limit, μ_T_ = μ_R_ + 1.96σ_BBR_ + ϵ, and μ_T_ = μ_R_ + 1.96σ_BBR_ + 2ϵ

Figure 7 illustrates the BBE type I error mean values with respect to the number of studied batches on three different estimations of the value of the Reference mean, showing mean value slightly greater than 5% with 3 batches in the Reference (type I error = 5.4%) at the bioequivalence limit. Type I error is continuously decreasing when the number of studies batches increases.

### Real Case Results: Challenge of the BBE Bioequivalence Limit

The objective of this section is to confirm the BEE bioequivalence limit initially set at 1.96. Although the simulations results highlighted reliable values on the pair (1 − *β*, *α*) with the prefixed BBE bioequivalence limit, it remains relevant to confirm this bioequivalence limit on real data. The 23 batches of Flonase and the 16 batches of Nasonex allowed to study the evolution of the TPR with respect to different acceptance criteria values for BBE in an equivalence context.

Table II summarizes the estimated BBE bioequivalence limits required to reach acceptable levels of TPR (80%, 85%, 90%, 95%). With more than 3 batches, the *θ* = 1.96 bioequivalence limit allowed to reach at least a level of 80% on the TPR. Moreover, the TPR level is greater than 90% with 7 batches.

**Table II:** BBE Acceptance Bioequivalence Limit to Reach True Positive Rates (TPR) of 80%, 85%, 90%, and 95%

	Product	*n*_BR_	TPR = 80%	TPR = 85%	TPR = 90%	TPR = 95%
DV50	Flonase®	3 (n_R = n_T = 30)	2.30	2.56	3.01	> 4
	Nasonex®	5 (n_R = n_T = 30)	1.86	2.01	2.24	2.61
	Nasonex®	7 (n_R = n_T = 30)	1.56	1.69	1.93	2.48
Area	Flonase®	3 (n_R = n_T = 30)	2.26	2.54	3.03	> 4
	Nasonex®	5 (n_R = n_T = 30)	1.85	2.00	2.20	2.55
	Nasonex®	7 (n_R = n_T = 30)	1.57	1.68	1.83	2.10

**Fig. 8:**
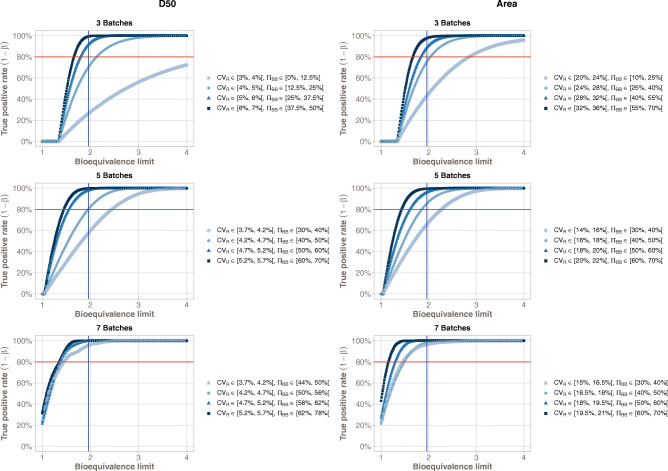
Bioequivalence true positive rates (power test profiles, *y*-axis) with respect to the relative acceptance bioequivalence limit. The study is done taking into account two parameters (Dv50 and Area, in columns) and three different number of batches (3: Flonase®, 5: Nasonex®, 7: Nasonex®). Each curve corresponds to different values of the couple (*CV*_R_, *π*_BB_)

Figure 8 illustrates the evolution of the TPR with respect to *θ*.The higher the pair (*CV*_R_, *π*_BB_) is (*i.e.*, higher the *σ*_BBR_ is), the lower the required BBE bioequivalence limit to reach an acceptable value of TPR is. These analyses show that the BBE bioequivalence limit fixed at *θ* = 1.96 is a reliable compromise for achieving a TPR level of at least 80%, especially with more than 3 batches.

### Real Case Results: True Positive Rate

As reported in Table II, the results observed in the real case application confirm those observed in the simulation results. Indeed, when the total variability of the Reference is low (around 5% for the DV50 criterion) the two state-of-the-art methods reached high true positive rates, with values greater than 99%. In this situation, the BBE equivalence test reached significantly lower true positive rates, depending on the between-batch variability (69% with *π*_BB_=25% and 84% with *π*_BB_ = 43%). On the other hand, with the Area parameter, exhibiting higher variability on the Reference, 27% and 18% for the Flonase and Nasonex products, the BBE method showed higher true positive rates than ABE and PBE (70% versus 14% and 60% for the Flonase and 85% versus 62 and 69% for Nasonex) (Table III).

**Table III:** Global Results on the Two Studied Real Cases, Namely the Application on the Flonase and Nasonex Commercial Products. Results are Separated into Two Parts, One for Each Parameter (D50 and Area) Characterizing the *In Vitro* Spray Performance. The Number of Batches Composing both the Reference and the Test products Are First Given Along with the Total Number of Products Composing the Reference and Test. Then, the Mean Total Variability of the Reference (E[*CV*_*R*_]) and Reference Between-Batch Variability (E[*π*_*BB*_]) Are Estimated. Finally, the True Positive Rates of each Bioequivalence Test Are Reported as a Percentage

	Product	*n*_*BR*_	*E[CV*_*R*_*]*	*E[π*_*BB*_*]*	True positive rate		
					ABE	PBE	BBE
DV50	Flonase®	3 (*n*_R_ = *n*_T_ = 30)	5%	25%	> 99%	> 99%	69%
	Nasonex®	5 (*n*_R_ = *n*_T_ = 30)	5%	43%	> 99%	> 99%	84%
Area	Flonase®	3 (*n*_R_ = *n*_T_ = 30)	27%	39%	14%	60%	70%
	Nasonex®	5 (*n*_R_ = *n*_T_ = 30)	18%	44%	62%	69%	85%

### Real Case Results: Concrete Examples

In order to illustrate the relative performance of the equivalence tests, concrete examples are given in this section.

#### Flonase®

**Fig. 9:**
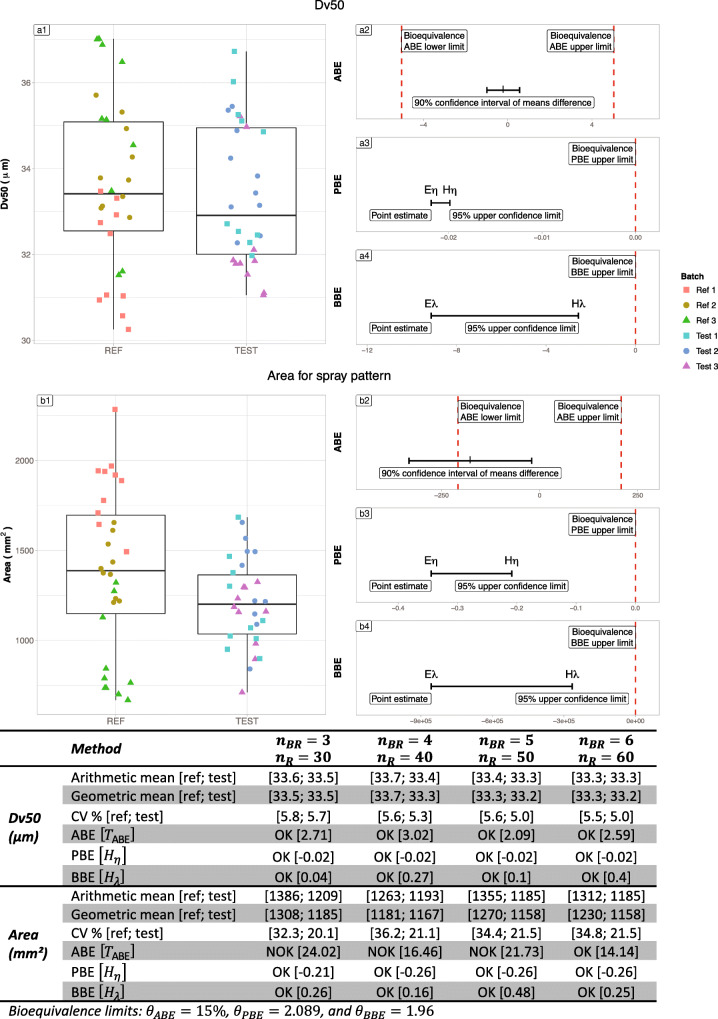
Illustration of the six batches of the Flonase® product Reference. Batches are separated into “REF” and “TEST” to test the three bioequivalence methods. A1 and B1 subfigures represent the products’ Dv50 and Area respectively. On the Right side, A2 and B2 represent ABE results for the Dv50 and Area parameters respectively, A3 and B3 PBE results and A4 and B4 BBE results. The table summarizes the bioequivalence test (average bioequivalence ABE, population bioequivalence (PBE), and between-batch bioequivalence BBE) results, using 3 (*N* = 30), 4 (*N* = 40), 5 (*N* = 50), and 6 (*N* = 60) batches for both the Reference and Test products

Table IV summarizes the results on the Flonase real case. The left part of Figure 9 exhibits raw data for Flonase® nasal spray batches, each composed of 10 samples. On the right side, the graphs represent the illustration of the bioequivalence test using the 3 statistical methods (ABE, PBE, and BBE). For the D50 criterion, the ratio between geometric means of Reference and Test is close to 1, the difference between the arithmetic means is low, and both variances are low (around 5%) whatever the number of batches. Thus, the three tests were able to recognize equivalence between Reference and Test. For the Area criterion, the ratio of geometric means is still close to 1 (from 0.90 to 0.99). However, the difference in arithmetic means is greater (around 10% of the Reference mean), the Reference variability is higher than the Test one (above 30% versus around 20% respectively). In such a situation, PBE and BBE were able to recognize the equivalence between Reference and Test from 3 batches while ABE needed an increased number of batches (6 batches).

**Table IV:** Summary of the bioequivalence tests results (Average Bioequivalence ABE, Population Bioequivalence (PBE), and Between−batch Bioequivalence BBE) for the Flonase® product

		*n*_BR_ = 3 *n*_R_ = 30	*n*_BR_ = 4 *n*_R_ = 28	*n*_BR_ = 5 *n*_R_ = 30	*n*_BR_ = 6 *n*_R_ = 30
Dv50 (*μm*)	Arithmetic mean [ref; test]	[33.59; 33.51]	[33.72; 33.39]	[33.39; 33.28]	[33.33; 33.28]
	Geometric mean [ref; test]	[33.54; 33.46]	[33.67; 33.34]	[33.33; 33.24]	[33.28; 33.24]
	Means difference [test − ref]	−0.082	−0.338	−0.109	−0.057
	Geometric means ratio [test/ref]	0.998	0.99	0.997	0.999
	Standard deviation [ref; test]	[1.95; 1.9]	[1.88; 1.77]	[1.87; 1.68]	[1.84; 1.68]
	sd difference [test − ref]	−0.053	−0.11	−0.199	−0.162
	CV% [ref; test]	[5.8; 5.66]	[5.59; 5.31]	[5.61; 5.03]	[5.51; 5.03]
	ABE [T_ABE_]	OK [2.72]	OK [3.05]	OK [2.1]	OK [2.62]
	PBE [H_η_]	OK [−0.02]	OK [−0.02]	OK [−0.02]	OK [−0.02]
	BBE [H_λ_]	OK [0.04]	OK [0.27]	OK [0.1]	OK [0.4]
Area (*mm*^2^)	Arithmetic mean [ref; test]	[1385.82; 1209.3]	[1263.36; 1193.47]	[1354.74; 1185.47]	[1311.65; 1185.47]
	Geometric mean [ref; test]	[1308.21; 1185.01]	[1180.91; 1166.94]	[1270.11; 1158.42]	[1230.48; 1158.42]
	Means difference [test − ref]	−176.523	−69.884	−169.275	−126.187
	Geometric means ratio [test/ref]	0.906	0.988	0.912	0.941
	Standard deviation [ref; test]	[448.01; 243.19]	[457.73; 252.34]	[466.07; 255.08]	[455.83; 255.08]
	sd difference [test − ref]	−204.826	−205.391	−210.993	−200.748
	CV% [ref; test]	[32.33; 20.11]	[36.23; 21.14]	[34.4; 21.52]	[34.75; 21.52]
	ABE [T_ABE_]	NOK [27.52]	NOK [17.42]	NOK [24.83]	OK [14.94]
	PBE [H_η_]	OK [−0.21]	OK [−0.26]	OK [−0.26]	OK [−0.26]
	BBE [H_λ_]	OK [0.26]	OK [0.16]	OK [0.48]	OK [0.25]

#### Nasonex®

**Fig. 10:**
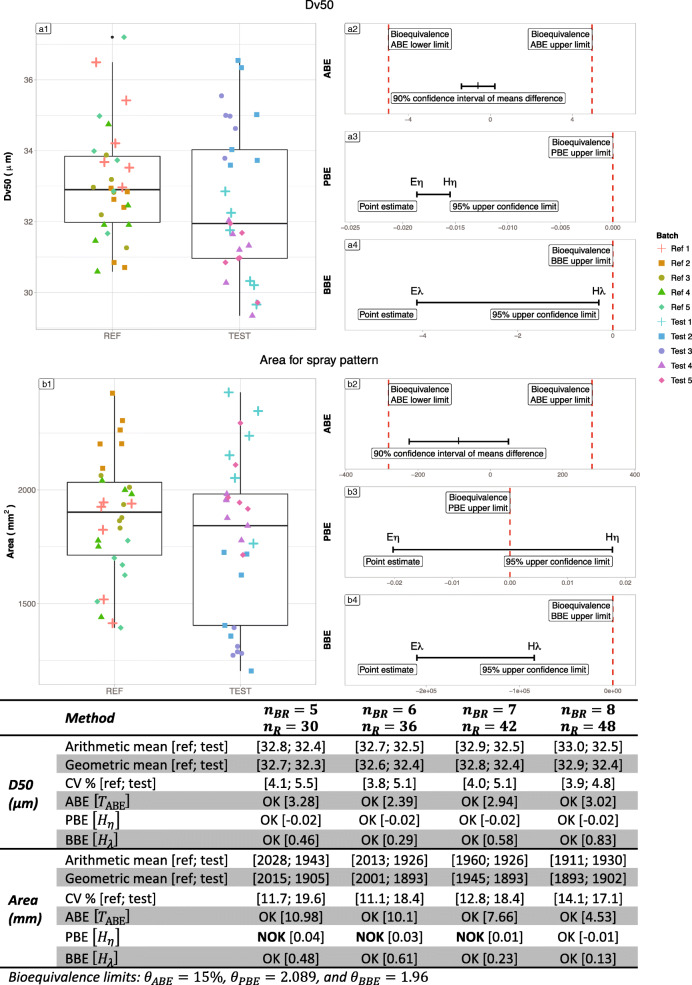
Illustration of the six batches of the Nasonex® product Reference. Batches are separated into “REF” and “TEST” to test the three bioequivalence methods. A1 and B1 subfigures represent the products’ Dv50 and Area respectively. On the Right side, A2 and B2 represent ABE results for the Dv50 and Area parameters respectively, A3 and B3 PBE results and A4 and B4 BBE results. The table summarizes the bioequivalence test (average bioequivalence ABE, population bioequivalence (PBE), and between-batch bioequivalence BBE) results, using 3 (N = 30), 4 (N = 40), 5 (N = 50), and 6 (N = 60) batches for both the Reference and Test products

Table V summarizes the results on the Nasonex real case. Figure 10 represents bioequivalence tests results for Nasonex® nasal spray batches each composed of 6 samples. For the D50 criterion, the ratio between geometric means of Reference and Test is close to 1, the difference between the arithmetic means is low, and both variances are low (around 5%) whatever the number of batches. Thus, the three tests were able to recognize equivalence between Reference and Test. For Area criterion, variance in Reference product is lower compared with Test product and remain moderate (below 20%). The boxplot in Fig. 8 reveal that two Test batches (tests 2 and 3) present lower values than the other, and lower than the Reference observations. The geometric means ratio increases with the number of batches (from 0.95 with 5 batches to 1.01 with 8 batches). This increase of the geometric means ratio, in combination with the increasing number of batches allowed PBE to accept bioequivalence from 8 batches.

**Table V:** Summary of the bioequivalence tests results (Average Bioequivalence ABE, Population Bioequivalence (PBE), and Between−batch Bioequivalence BBE) for the Nasonex® product

		*n*_BR_ = 5 *n*_R_ = 30	*n*_BR_ = 6 *n*_R_ = 30	*n*_BR_ = 7 *n*_R_ = 28	*n*_BR_ = 8 *n*_R_ = 32
Dv50 (*μm*)	Arithmetic mean [ref; test]	[32.76; 32.36]	[32.67; 32.46]	[32.86; 32.46]	[32.95; 32.46]
	Geometric mean [ref; test]	[32.73; 32.31]	[32.64; 32.42]	[32.84; 32.42]	[32.93; 32.42]
	Means difference [test − ref]	−0.398	−0.204	−0.402	−0.492
	Geometric means ratio [test/ref]	0.987	0.993	0.987	0.985
	Standard deviation [ref; test]	[1.33; 1.77]	[1.25; 1.65]	[1.32; 1.65]	[1.29; 1.56]
	sd difference [test − ref]	0.443	0.395	0.333	0.264
	CV% [ref; test]	[4.06; 5.48]	[3.84; 5.08]	[4; 5.08]	[3.92; 4.8]
	ABE [T_ABE_]	OK [3.32]	OK [2.4]	OK [2.99]	OK [3.08]
	PBE [H_η_]	OK [−0.02]	OK [−0.02]	OK [−0.02]	OK [−0.02]
	BBE [H_λ_]	OK [0.46]	OK [0.29]	OK [0.58]	OK [0.83]
Area (*mm*^2^)	Arithmetic mean [ref; test]	[2028.25; 1942.89]	[2012.99; 1926.2]	[1960.46; 1926.2]	[1911.4; 1930.4]
	Geometric mean [ref; test]	[2015.04; 1905.05]	[2001.36; 1893.42]	[1944.65; 1893.42]	[1892.83; 1901.86]
	Means difference [test − ref]	−85.361	−86.788	−34.259	19.001
	Geometric means ratio [test/ref]	0.945	0.946	0.974	1.005
	Standard deviation [ref; test]	[237.85; 380.41]	[222.84; 354.05]	[251.47; 354.05]	[269.89; 329.25]
	sd difference [test − ref]	142.561	131.209	102.587	59.365
	CV% [ref; test]	[11.73; 19.58]	[11.07; 18.38]	[12.83; 18.38]	[14.12; 17.06]
	ABE [T_ABE_]	OK [11.46]	OK [10.55]	OK [7.9]	OK [6.5]
	PBE [H_η_]	NOK [0.04]	NOK [0.03]	NOK [0.01]	OK [−0.01]
	BBE [H_λ_]	OK [0.48]	OK [0.61]	OK [0.23]	OK [0.13]

## DISCUSSION

In this work, an alternative bioequivalence method is proposed to mitigate the two state-of-the-art methods (PBE and ABE) limitations. This method, named between-batch bioequivalence (BBE), is based on the comparison between the mean difference (Reference − Test) and the Reference between-batch variability. The theoretical justification of this approach was established through the derivation of the relevant statistic test (T_*BBE*_) which follows a noncentral Student’s *T* distribution. An exact procedure was developed to implement the BBE approach. In addition, this work also introduces an illustrative method (BBE_CI_) to compute BBE through the confidence interval estimation. This illustrative method brings a simple and robust way to quickly illustrate the BBE results. The BBE performance was evaluated through its true positive rate estimation on simulated data. Furthermore, these true positive rates were compared with the ones of ABE and PBE. In a second step, the false positive rate of the BBE method was evaluated on the simulated data. Finally, to illustrate the advantages and drawbacks of BBE compared with ABE and PBE, the three methods were applied on two commercial nasal spray products and their ability to demonstrate equivalence was evaluated.

Regulatory authorities in the United States (FDA) and the European Union (EMA) recommend the application of respectively PBE and ABE to assess equivalence between a generic and a reference drug. However, these two methods do not take into account the repartition of both the Reference and Test products into batches. More importantly, they do not consider the variability between the different batches and what may be a strong limitation, as reported by several studies (2,9,21,27,28). More recently, the International Pharmaceutical Aerosol Consortium on Regulation and Science (IPAC-RS) PBE working group evaluated the impact of increasing the Reference batches number and the consideration of between-batch variability in ABE and PBE (37). This report highlighted the beneficial effect of increasing the number of sampled batches and considering the between-batch variability in bioequivalence tests (both ABE and PBE) which increase the true positive rate and decrease the false positive rate. However, considering that the between-batch variability in ABE and PBE did not overpass the inherent limitations at high CV_R_ values of these methods, *i.e.*, the ABE true positive rate declined and PBE increased permissiveness to a large difference between Reference and Test means.

The differences observed between the true positive rates of the three bioequivalence methods can be explained by their specific mathematical definitions and thus their biological equivalence definitions. Indeed, ABE only considers the product variabilities to construct the confidence interval of the mean difference. Thus, ABE type II error increases when the variability and consequently between-batch variability increases. On the other side, PBE considers the variability difference between Reference and Test and compares the mean difference (*μ*_T_ − *μ*_R_) with the Reference variability. Thus, PBE type II error decreases when the Reference variability increases, especially when the Reference variability is greater than the Test variability. In opposition, BBE compares the mean difference (*μ*_T_ − *μ*_R_) with the Reference between-Batch variability, which is usually related to the total Reference variability. Thus, BBE type II error decreases when the Reference between-Batch variability increases.

As expected, the higher the Reference variability and especially the between-batch variability, the greater the BBE true positive rate. Even with a relatively low between-batch variability (*e.g.*, 20%), the BBE TPR is higher than 80% with 5 batches and higher than 90% with 7 batches, from a CV_R_ of 6%. However, under a CV_R_ of 6%, the BBE TPR decreases and thus, BBE seems less appropriate. The ABE TPR increases with the number of Reference batches considered, even with the same total number of samples (*e.g.*, considering 30 samples, ABE TPR > 80% for CV_R_ up to 10% and 3 batches whereas with 10 batches CV_R_ goes up to 15%). However, a big loss of ABE statistical strength is observed when the CV_R_ increase. In other words, ABE is less appropriate from a CV_R_ of 10% and 3 batches (TPR < 80%).

A high PBE TPR is observed for CV_R_ up to 10% and high CV_R_ from 30%. This behavior can be explained by the PBE formula itself (29): the denominator of the PBE statistic takes the maximum between the Reference variability and 10%, explaining the inflection point observed in the TPR curves. After this 10% threshold, the PBE statistic inversely depends on the CV_R_ value. Thus, the PBE test will be more permissive to high mean differences for the highest Reference variabilities (11). Between these two CV_R_ values (10% to 30%), the PBE TPR goes from 50 to 80%, depending on the number of batches in the Reference: the higher the batches number, the higher the TPR.

This study suggests that the number of batches used to evaluate bioequivalence between a generic product and its Reference should be increased. There is a strong dependence between this number of Reference batches and the true positive rate that can be explained by a better estimation of the mean and the variability of the population by considering more batches. The US FDA (17) recommends a minimum of 30 samples (*i.e.*, 3 batches of 10 samples). From this study, the recommendation would be to use at least 5 batches of 6 units, keeping the total amount of samples at 30. This recommendation is aligned with the recent report of Chen *et al.* suggesting to increase the number of sampled batches rather than the total number of measurements (37). A goodness-of-fit study was performed on the real data to validate that the estimates of the means are sufficiently accurate with 6 units per batch in comparison with 10 units per batch. This study is detailed in the supplementary materials. At a constant sample size per product (*n*_R_ = *n*_T_ =  30), it seems more appropriate to take 5 batches of 6 units rather than 3 batches of 10 units. In summary, increasing the batches number may constitute a valuable alternative approach to the total sample size increase. This approach is more compatible to the context of a generic drug development by resulting in a better characterization of both the Reference and Test populations without increasing the total number of samples.

Considering the batch factor in the bioequivalence test seems essential to better evaluate the between-batch variability. As previously reported in several studies [9,21,39], this lack of consideration of the between-batch variability (not taken into account in the PBE and ABE formula) can lead to a true positive rate decrease for non-negligible values. While this should not induce a higher risk for the customer, this may lead to increase the risk to erroneously reject equivalence and thus indirectly the development costs. From a different perspective, regulatory authorities recommend increasing the sample size when the true positive rate is low (4,5,25,26). However, this could induce a counterproductive effect as demonstrated in Chen *et al.* (37), increasing the number of measurements within a batch inflates the false positive rate when the between-batch variability is high and the number of batches is low.

The BBE method is based on a mixed effect model formula, taking the batch into account as a nested factor. Thus, the batch factor and the between-batch variability are considered in the BBE formula.

Overall, the BBE true positive rate analysis highlight high and stable values. Indeed, from a Reference variability of 6%, the BBE TPR remains stable over the studied range of Reference variability values. Furthermore, with at least 5 batches, TPR is greater than 80% (when CV_R_ ≥ 6%). Thus, this method allows to reach reasonable performance without needing to increase the total sample size. The benefits of BBE have been illustrated through a real case study of two commercially available nasal sprays using bioequivalence assumptions. In contrast to ABE and PBE, BBE was able to prove bioequivalence in all cases with 30 samples, whereas additional experimental measurements have been conducted by increasing the samples up to 60 samples in the case of ABE for Flonase and up to 48 samples in the case of PBE for Nasonex. This real case application highlighted the strengths and weaknesses of each method. ABE is strongly dependent on within-product variability because this method is performed through the confidence interval of the mean difference. Furthermore, the Welch-Satterthwaite correction of the degrees of freedom expands the confidence interval under heteroscedasticity, which is quite often observed in real cases. On the other side, PBE is strongly dependent on the difference between the Reference and Test variability, promoting situations where Test variability is lower than the Reference variability, but penalized situations with lower Reference within-product variability. By considering between-batch variability, BBE is useful when the Reference between-batch variability is a non-negligible part of the Reference within-product variability.

One of the main results of this study was the identification of ranges, in terms of total and between-batch variability and of batch numbers, where the BBE TPR was higher than the two other methods. Indeed, results showed that whatever the total number of batches in the Reference, the BBE TRP is always higher than ABE when CV_R_ is greater than 15%, this threshold being reduced when the between-batch variability increases, going from CV_R_ = 15% when the between-batch variability represent 20% of the total variability to CV_R_ = 10% when the between-batch variability increases to 80% of the total variability. When comparing BBE with PBE, the threshold seems independent to the between-batch variability and the number of batches in the Reference. However, the true positive rate gain itself depends on the number of batches going from a minimal value of 15% with 3 batches to 20% with 10 batches.

While the BBE method showed strengths and advantages as compared with the ABE and PBE methods, there are also some limitations. First of all, the BBE required at least 5 batches to reach a suitable performance. Another limitation of the BBE method came from its dependence on the total variability on the Reference. Particularly, the method showed weak true positive rates for the lowest values of CV_R_ (CV_R_ ≤ 6%). This observation emphasizes that the BBE method may not be appropriate in low variability situations, especially when few batches are considered. Regarding the bioequivalence limits, FDA and EMA have defined bioequivalence limits in the guidance (15,22). The BBE bioequivalence limit has been defined at 1.96 and evaluated through the simulation studies described above. However, this value still requires an evaluation by regulatory authorities. Finally, the BBE method assumes equality in Reference and Test between-batch variances. Indeed, only the Reference between-batch variability is considered in the equation of the model. Even though this assumption may appear as a limitation of BBE, the Reference between-batch variability is quite often in real life higher than that of the Test, because of a longer time interval between batch productions. Thus, the Test between-batch variability is underestimated leading a greater stringency of the method and avoids false positives (*i.e.*, the inflation of the type I error). In other words, BBE will avoid considering the two products as equivalent if the Test between-batch variability is higher than that of the Reference. Also due to the longer time interval between batch productions, the Reference within-product variability is mainly explained by the between-batch variability, or at least the between-batch variability will be high enough to have statistical reliability on BBE.

As a first perspective, the dependency on the number of batches that both ABE and the PBE TPR exhibit asks whether they are possibly dependent on the false positive rate. Indeed, this aspect was partially covered in the IPAC-RS PBE working group. A more systematic study approach may be useful to ensure the final consumer safety. In this study, the real case application was limited to *in vitro* nasal spray *in vitro* bioequivalence. We recommend conducting further bioequivalence studies to evaluate the method and prove its relevance for both *in vivo* and *in vitro* bioequivalence testing. Further studies should be conducted to explore the PBE TPR under heteroscedasticity. Last but not least, it could be interesting to set up a protocol that combines the advantages of each method. In that goal, a unique criterion function of the variability (between-batch and within-product) should be defined to characterize the validity range of each method.

## CONCLUSION

The purpose of this work was to propose the development of an alternative statistical method to evaluate equivalence. In opposition to ABE and PBE, the BBE method considers the between-batch variability.

Simulation and real data studies proved the robustness of BBE compared with ABE and PBE, especially in a non-zero between-batch variability context. Moreover, BBE does not require to increase the number of samples in well-known cases where ABE and PBE have lower TPR.

This work highlighted the BBE prerequisites, namely at least five batches per product (Reference and Test) and a relative standard deviation on the Reference product (CV_R_) greater than 6%. BBE computation, described in the section “BBE computation,” can be accomplished with elementary calculations or with the following web application [40].

Thus, BBE may be of particular interest to optimize the generic development in both *in vitro* and *in vivo* contexts.

## Electronic Supplementary Material

ESM 1 (DOCX 269 kb)

## References

[1] Chen ML, Blume H, Beuerle G, Davit B, Mehta M, Potthast H (2018). The global bioequivalence harmonization initiative: summary report for EUFEPS international conference. Eur J Pharm Sci.

[2] Lee SL, Saluja B, García-Arieta A, Santos GML, Li Y, Lu S, Hou S, Rebello J, Vaidya A, Gogtay J, Purandare S, Lyapustina S (2015). Regulatory considerations for approval of generic inhalation drug products in the US, EU, Brazil, China, and India. AAPS J.

[3] Lu D, Lee SL, Lionberger RA, Choi S, Adams W, Caramenico HN (2015). International guidelines for bioequivalence of locally acting orally inhaled drug products: similarities and differences. AAPS J.

[4] Souza R. Statistical Analysis in bioequivalence studies. Biostat Biometrics Open Access J. Juniper Publishers; 2017;3.

[5] Ocanã J, Sánchez O. MP, Sánchez A, Carrasco JL. On equivalence and bioequivalence testing. SORT. 2008.

[6] Nandakumar SP. Statistical procedures for bioequivalence analysis. Western Michigan University; 2009.

[7] Fearn T, Chow S-C, Liu J-P. Design and analysis of bioavailability and bioequivalence studies. [Internet]. Chapman and Hall/CRC. 2008. Available from: https://www.crcpress.com/Design-and-Analysis-of-Bioavailability-and-Bioequivalence-Studies/Chow-Liu/p/book/9781584886686.

[8] Sarfaraz N. Handbook of bioequivalence testing. CRC Press 2017.

[9] Morgan B, Chen S, Christopher D, Långström G, Wiggenhorn C, Burmeister Getz E (2018). Performance of the population bioequivalence (PBE) statistical test using an IPAC-RS database of delivered dose from metered dose inhalers. AAPS PharmSciTech.

[10] FDA, CDER. Draft guidance: average, population, and individual approaches to establishing bioequivalence. 1999.

[11] García-Arieta A, Gordon J. Bioequivalence requirements in the European Union: Critical discussion. AAPS J. 2012:738–48. 10.1208/s12248-012-9382-1 PMC3475855 22826032

[12] Santos C, Marco G, Nagao L, Castro E, Chesworth T (2018). European regulatory developments for orally inhaled and nasal drug products. AAPS PharmSciTech.

[13] Shuirmann DJ (1987). A comparison of the two one-sided tests procedure and the power. J Pharmacokinet Biopharm.

[14] US Food and Drug administration. Draft guidance on budesonide [Internet]. 2016. Available from: http://www.fda.gov/downloads/Drugs/GuidanceComplianceRegulatoryInformation/Guidances/UCM319977.pdf.

[15] US Food and Drug Administration. Statistical Approachaes to establishing bioequivalence [Internet]. 2001. Available from: http://www.fda.gov/cder/guidance/index.htm.

[16] US Food and Drug Administration. Draft guidance on fluticasone propionate. 2019.

[17] US Food and Drug Administration. Statistical information from the June 1999 draft guidance and statistical information for in vitro bioequivalence data posted on 1999.

[18] US Food and Drug Administration. Bioavailability and bioequivalence studies for nasal aerosols and nasal sprays for local action [Internet]. 2003. Available from: http://www.fda.gov/cder/guidance/index.htm.

[19] US Food and Drug Administration. Draft guidance on mometasone furoate monohydrate. 2019.

[20] Trows S, Wuchner K, Spycher R, Steckel H. Analytical challenges and regulatory requirements for nasal drug products in Europe and the U.S. Pharmaceutics. MDPI AG; 2014. p. 195–219. 10.3390/pharmaceutics6020195 PMC4085595 24732068

[21] Morgan B, Strickland H (2014). Performance properties of the population bioequivalence approach for in vitro delivered dose for orally inhaled respiratory products. AAPS J.

[22] Chow S-C (2014). Bioavailability and bioequivalence in drug development. Wiley Interdiscip Rev Comput Stat.

[23] Grmaš J, Lužar-Stiffler V, Dreu R, Injac R (2019). A novel simulation-based approach for comparing the population against average bioequivalence statistical test for the evaluation of nasal spray products on spray pattern and droplet size distribution parameters. AAPS PharmSciTech.

[24] Rani S, Pargal A. Bioequivalence: an overview of statistical concepts. Indian J Pharmacol. 2004;

[25] Li B V., Jin F, Lee SL, Bai T, Chowdhury B, Caramenico HT, *et al.* Bioequivalence for locally acting nasal spray and nasal aerosol products: standard development and generic approval. AAPS J. 2013. p. 875–83. 10.1208/s12248-013-9494-2 PMC3691440 23686396

[26] Burmeister Getz E, Carroll KJ, Jones B, Benet LZ (2016). Batch-to-batch pharmacokinetic variability confounds current bioequivalence regulations: a dry powder inhaler randomized clinical trial. Clin Pharmacol Ther.

[27] Burmeister Getz E, Carroll KJ, Mielke J, Benet LZ, Jones B (2017). Between-batch pharmacokinetic variability inflates type I error rate in conventional bioequivalence trials: a randomized Advair Diskus clinical trial. Clin Pharmacol Ther Nature Publishing Group.

[28] US Food and Drug Administration. Draft Guidance on Budesonide. 2012.

[29] Liandra S, Petitcolas C, Jonathan B. Method for testing bioequivalence of a distribution device. France. 2020:1–31.

[30] Hedges L V. Distribution theory for glass’s estimator of effect size and related estimators. J Educ Stat [Internet]. 1981;6:107. Available from: https://www.jstor.org/stable/1164588?origin=crossref.

[31] US Food and Drug Administration. Guidance for industry - nasal spray and inhalation solution, suspension, and spray drug products - chemistry, manufacturing, and controls documentation. 2002.

[32] Inthavong K, Fung MC, Yang W, Tu J (2015). Measurements of droplet size distribution and analysis of nasal spray atomization from different actuation pressure. J Aerosol Med Pulm Drug Deliv.

[33] Inthavong K, Fung MC, Tong X, Yang W, Tu J (2014). High resolution visualization and analysis of nasal spray drug delivery. Pharm Res.

[34] Grmas J, Rok D, Rade I (2019). Analytical challenges of spray pattern method development for purposes of in vitro bioequivalence testing in the case of a nasal spray product. J Aerosol Med Pulm Drug Deliv.

[35] R Core Team. R: a language and environment for statistical computing [Internet]. Vienna, Austria; 2019. Available from: https://www.r-project.org/.

[36] Chen S, Morgan B, Beresford H, Burmeister Getz E, Christopher D, Långström G, et al. Performance of the population bioequivalence (PBE) statistical test with impactor sized mass data. AAPS PharmSciTech AAPS PharmSciTech; 2019;20. 10.1208/s12249-019-1507-8 31444601

[37] European Medicines Agency. Guideline on the requirements for clinical documentation for orally inhaled products (Oip) including the requirements for demonstration of therapeutic equivalence between two inhaled products for use in the treatment of asthma and chronic obstructive pulm. Pdf [Internet]. 2009;1–26. Available from: http://www.ema.europa.eu/docs/en_GB/document_library/Scientific_guideline/2009/09/WC500003504.pdf.

