# Supplementary material for: Between-Batch Bioequivalence (BBE): a Statistical Test to Evaluate In Vitro Bioequivalence Considering the Between-Batch Variability
Source: AAPS J. 2020 Sep 10;22(5):119. doi: 10.1208/s12248-020-00486-5 (PMC7651657; doi:10.1208/s12248-020-00486-5)
Supplement: Supplementary file 1 — (DOCX 269 kb) [file 12248_2020_486_MOESM1_ESM.docx]

# Supplementary Materials


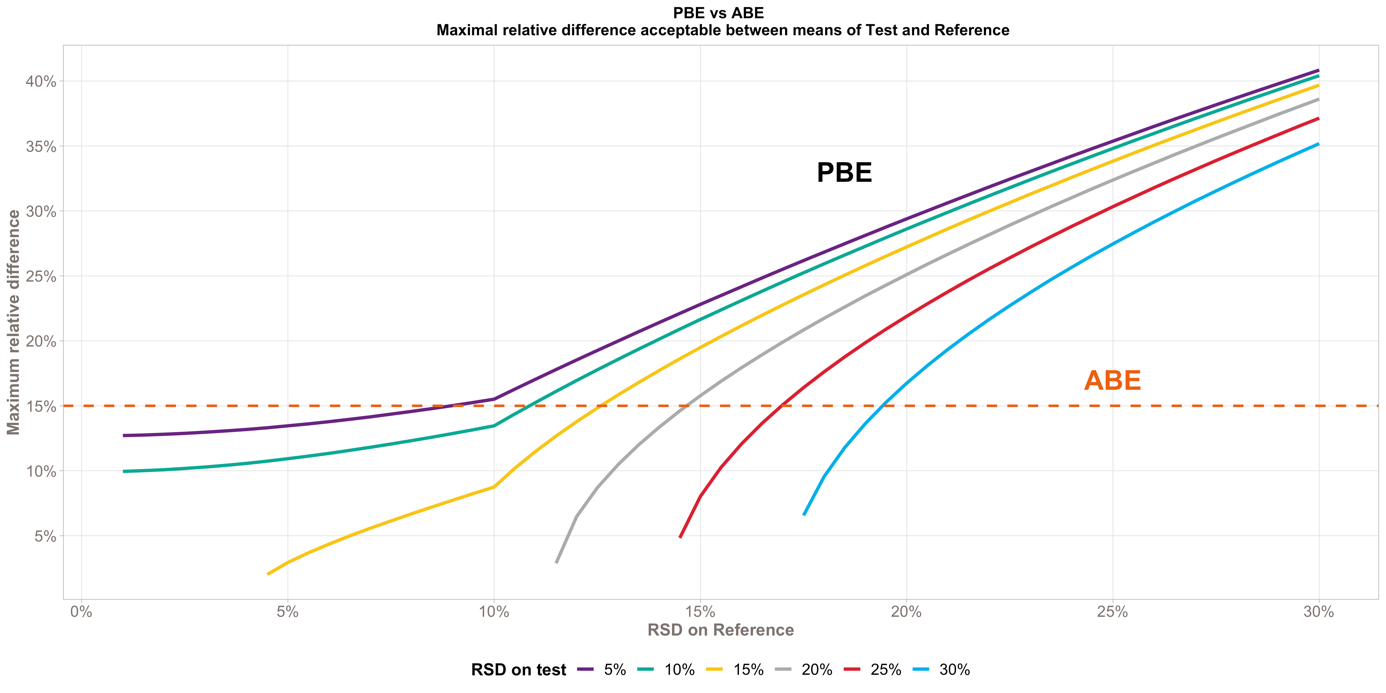


Figure S1:

This graph illustrates the theories of ABE and PBE. The x axis represents the relative standard deviation of the Reference. The y axis represents the maximum delta acceptable to pass the bioequivalence (in other words it corresponds to the maximum ratio between means of test and reference). For ABE, as this is only a means comparison, whatever the relative standard deviations are, the maximum delta acceptable is a straight line. The usual limit is 15%. For PBE, the results are strongly dependent of the variabilities of the results and much more from reference. The illustration is done through 2 examples using the same variability for the test fixed at 10% (this means the orange curve). The first example with a variability of the reference at 5% gives a delta maximum for the means of 10% which is lower than ABE which is at 15%. In this case, PBE is less permissive than ABE. The second one with a variability of the reference at 20% gives a delta max for the means around 28% which is much higher than ABE which is still at 15%. In this case PBE is more permissive than ABE.

|  |  | ***3 batches*** | | | | | | | ***5 batches*** | | | | | | | ***7 batches*** | | | | | | | ***10 batches*** | | | | | | |
| --- | --- | --- | --- | --- | --- | --- | --- | --- | --- | --- | --- | --- | --- | --- | --- | --- | --- | --- | --- | --- | --- | --- | --- | --- | --- | --- | --- | --- | --- |
| Total var  BB var | | *5%* | *10%* | *15%* | *20%* | *25%* | *30%* | *35%* | *5%* | *10%* | *15%* | *20%* | *25%* | *30%* | *35%* | *5%* | *10%* | *15%* | *20%* | *25%* | *30%* | *35%* | *5%* | *10%* | *15%* | *20%* | *25%* | *30%* | *35%* |
| 10% | ABE | 1.00 | 1.00 | 0.93 | 0.69 | 0.42 | 0.18 | 0.05 | 1.00 | 1.00 | 0.99 | 0.85 | 0.55 | 0.25 | 0.07 | 1.00 | 1.00 | 1.00 | 0.90 | 0.60 | 0.25 | 0.06 | 1.00 | 1.00 | 1.00 | 0.96 | 0.73 | 0.36 | 0.10 |
|  | PBE | 1.00 | 0.96 | 0.82 | 0.81 | 0.78 | 0.75 | 0.74 | 1.00 | 0.98 | 0.86 | 0.83 | 0.81 | 0.78 | 0.74 | 1.00 | 0.97 | 0.84 | 0.82 | 0.79 | 0.77 | 0.74 | 1.00 | 0.98 | 0.85 | 0.84 | 0.81 | 0.79 | 0.76 |
|  | BBE | 0.67 | 0.67 | 0.68 | 0.68 | **0.67** | **0.68** | **0.67** | 0.84 | 0.84 | 0.84 | 0.84 | **0.84** | **0.84** | **0.83** | 0.92 | 0.92 | 0.92 | **0.92** | **0.92** | **0.93** | **0.93** | 0.98 | 0.98 | 0.98 | **0.98** | **0.98** | **0.98** | **0.98** |
| 20% | ABE | 1.00 | 0.98 | 0.81 | 0.54 | 0.31 | 0.13 | 0.05 | 1.00 | 1.00 | 0.93 | 0.69 | 0.42 | 0.19 | 0.05 | 1.00 | 1.00 | 0.97 | 0.76 | 0.45 | 0.18 | 0.04 | 1.00 | 1.00 | 0.99 | 0.87 | 0.58 | 0.27 | 0.07 |
|  | PBE | 1.00 | 0.91 | 0.75 | 0.75 | 0.72 | 0.71 | 0.70 | 1.00 | 0.97 | 0.82 | 0.81 | 0.77 | 0.76 | 0.73 | 1.00 | 0.97 | 0.83 | 0.80 | 0.77 | 0.75 | 0.73 | 1.00 | 0.98 | 0.85 | 0.83 | 0.81 | 0.78 | 0.75 |
|  | BBE | 0.67 | 0.68 | 0.67 | **0.68** | **0.68** | **0.68** | **0.68** | 0.84 | 0.84 | 0.83 | **0.84** | **0.83** | **0.84** | **0.83** | 0.93 | 0.92 | 0.93 | **0.92** | **0.93** | **0.92** | **0.93** | 0.98 | 0.98 | 0.98 | **0.98** | **0.98** | **0.98** | **0.98** |
| 30% | ABE | 1.00 | 0.95 | 0.70 | 0.45 | 0.25 | 0.12 | 0.05 | 1.00 | 0.99 | 0.86 | 0.59 | 0.34 | 0.15 | 0.05 | 1.00 | 1.00 | 0.92 | 0.67 | 0.38 | 0.15 | 0.04 | 1.00 | 1.00 | 0.97 | 0.79 | 0.49 | 0.22 | 0.06 |
|  | PBE | 1.00 | 0.85 | 0.70 | 0.68 | 0.67 | 0.66 | 0.66 | 1.00 | 0.93 | 0.78 | 0.77 | 0.74 | 0.73 | 0.71 | 1.00 | 0.96 | 0.80 | 0.78 | 0.76 | 0.73 | 0.72 | 1.00 | 0.98 | 0.85 | 0.82 | 0.80 | 0.77 | 0.76 |
|  | BBE | 0.68 | 0.67 | 0.68 | **0.68** | **0.68** | **0.67** | **0.68** | 0.84 | 0.83 | 0.83 | **0.83** | **0.83** | **0.84** | **0.84** | 0.93 | 0.93 | **0.93** | **0.92** | **0.93** | **0.92** | **0.93** | 0.98 | 0.98 | **0.98** | **0.98** | **0.98** | **0.98** | **0.98** |
| 40% | ABE | 1.00 | 0.92 | 0.63 | 0.39 | 0.22 | 0.11 | 0.05 | 1.00 | 0.98 | 0.80 | 0.53 | 0.30 | 0.15 | 0.05 | 1.00 | 1.00 | 0.87 | 0.60 | 0.34 | 0.13 | 0.04 | 1.00 | 1.00 | 0.94 | 0.73 | 0.44 | 0.19 | 0.06 |
|  | PBE | 1.00 | 0.79 | 0.62 | 0.62 | 0.63 | 0.62 | 0.63 | 1.00 | 0.90 | 0.73 | 0.72 | 0.71 | 0.69 | 0.69 | 1.00 | 0.93 | 0.78 | 0.76 | 0.73 | 0.71 | 0.70 | 1.00 | 0.97 | 0.83 | 0.81 | 0.79 | 0.75 | 0.74 |
|  | BBE | 0.67 | 0.67 | **0.66** | **0.67** | **0.68** | **0.68** | **0.68** | 0.83 | 0.84 | **0.83** | **0.83** | **0.84** | **0.84** | **0.84** | 0.93 | 0.92 | **0.93** | **0.92** | **0.92** | **0.93** | **0.93** | 0.98 | 0.98 | **0.97** | **0.98** | **0.98** | **0.98** | **0.98** |
| 50% | ABE | 1.00 | 0.87 | 0.59 | 0.36 | 0.20 | 0.11 | 0.06 | 1.00 | 0.97 | 0.75 | 0.49 | 0.27 | 0.13 | 0.05 | 1.00 | 0.99 | 0.83 | 0.55 | 0.30 | 0.12 | 0.03 | 1.00 | 1.00 | 0.91 | 0.66 | 0.39 | 0.18 | 0.05 |
|  | PBE | 1.00 | 0.75 | 0.58 | 0.58 | 0.58 | 0.58 | 0.58 | 1.00 | 0.86 | 0.69 | 0.68 | 0.68 | 0.67 | 0.66 | 1.00 | 0.91 | 0.75 | 0.72 | 0.70 | 0.70 | 0.68 | 1.00 | 0.95 | 0.81 | 0.78 | 0.77 | 0.75 | 0.72 |
|  | BBE | 0.68 | 0.68 | **0.68** | **0.67** | **0.67** | **0.67** | **0.68** | 0.83 | 0.83 | **0.84** | **0.83** | **0.83** | **0.84** | **0.84** | 0.92 | 0.93 | **0.93** | **0.92** | **0.93** | **0.93** | **0.93** | 0.98 | 0.98 | **0.98** | **0.98** | **0.98** | **0.98** | **0.98** |
| 60% | ABE | 1.00 | 0.84 | 0.56 | 0.33 | 0.19 | 0.11 | 0.05 | 1.00 | 0.95 | 0.71 | 0.45 | 0.24 | 0.12 | 0.05 | 1.00 | 0.98 | 0.79 | 0.51 | 0.28 | 0.12 | 0.04 | 1.00 | 1.00 | 0.88 | 0.62 | 0.36 | 0.16 | 0.06 |
|  | PBE | 1.00 | 0.71 | 0.55 | 0.53 | 0.54 | 0.55 | 0.55 | 1.00 | 0.83 | 0.65 | 0.65 | 0.64 | 0.63 | 0.63 | 1.00 | 0.88 | 0.69 | 0.70 | 0.68 | 0.67 | 0.66 | 1.00 | 0.93 | 0.78 | 0.77 | 0.74 | 0.72 | 0.70 |
|  | BBE | 0.68 | 0.68 | **0.68** | **0.68** | **0.68** | **0.68** | **0.68** | 0.83 | 0.84 | **0.84** | **0.83** | **0.84** | **0.84** | **0.84** | 0.93 | 0.93 | **0.93** | **0.93** | **0.93** | **0.93** | **0.93** | 0.98 | 0.98 | **0.98** | **0.98** | **0.98** | **0.98** | **0.98** |
| 70% | ABE | 1.00 | 0.81 | 0.51 | 0.31 | 0.17 | 0.10 | 0.06 | 1.00 | 0.93 | 0.67 | 0.41 | 0.23 | 0.12 | 0.06 | 1.00 | 0.97 | 0.75 | 0.48 | 0.26 | 0.11 | 0.04 | 1.00 | 0.99 | 0.85 | 0.59 | 0.34 | 0.15 | 0.05 |
|  | PBE | 0.99 | 0.68 | 0.50 | 0.50 | 0.51 | 0.50 | 0.52 | 1.00 | 0.79 | 0.62 | 0.60 | 0.61 | 0.61 | 0.61 | 1.00 | 0.84 | 0.67 | 0.66 | 0.66 | 0.65 | 0.64 | 1.00 | 0.91 | 0.74 | 0.74 | 0.72 | 0.71 | 0.69 |
|  | BBE | 0.68 | 0.68 | **0.67** | **0.67** | **0.68** | **0.67** | **0.67** | 0.84 | 0.83 | **0.84** | **0.83** | **0.84** | **0.83** | **0.84** | 0.93 | 0.92 | **0.92** | **0.92** | **0.92** | **0.93** | **0.92** | 0.98 | 0.98 | **0.98** | **0.98** | **0.98** | **0.98** | **0.98** |
| 80% | ABE | 1.00 | 0.77 | 0.48 | 0.29 | 0.17 | 0.10 | 0.06 | 1.00 | 0.91 | 0.64 | 0.39 | 0.23 | 0.12 | 0.06 | 1.00 | 0.96 | 0.72 | 0.46 | 0.23 | 0.11 | 0.04 | 1.00 | 0.99 | 0.82 | 0.56 | 0.33 | 0.14 | 0.06 |
|  | PBE | 0.99 | 0.65 | 0.49 | 0.47 | 0.48 | 0.48 | 0.49 | 1.00 | 0.76 | 0.59 | 0.57 | 0.58 | 0.57 | 0.58 | 1.00 | 0.82 | 0.63 | 0.63 | 0.62 | 0.63 | 0.62 | 1.00 | 0.89 | 0.72 | 0.70 | 0.69 | 0.67 | 0.67 |
|  | BBE | 0.67 | 0.68 | **0.68** | **0.67** | **0.67** | **0.68** | **0.67** | 0.83 | 0.83 | **0.84** | **0.83** | **0.83** | **0.83** | **0.83** | 0.92 | 0.92 | **0.92** | **0.92** | **0.92** | **0.93** | **0.93** | 0.98 | 0.98 | **0.98** | **0.98** | **0.98** | **0.98** | **0.98** |
| 90% | ABE | 0.99 | 0.75 | 0.46 | 0.29 | 0.17 | 0.10 | 0.06 | 1.00 | 0.89 | 0.62 | 0.37 | 0.22 | 0.11 | 0.07 | 1.00 | 0.95 | 0.70 | 0.42 | 0.23 | 0.11 | 0.05 | 1.00 | 0.98 | 0.80 | 0.53 | 0.30 | 0.15 | 0.06 |
|  | PBE | 0.98 | 0.63 | 0.49 | 0.45 | 0.45 | 0.45 | 0.46 | 1.00 | 0.74 | 0.57 | 0.55 | 0.56 | 0.54 | 0.56 | 1.00 | 0.80 | 0.62 | 0.60 | 0.60 | 0.60 | 0.60 | 1.00 | 0.87 | 0.69 | 0.68 | 0.66 | 0.66 | 0.65 |
|  | BBE | 0.68 | 0.67 | **0.68** | **0.68** | **0.67** | **0.68** | **0.68** | 0.84 | 0.84 | **0.84** | **0.83** | **0.84** | **0.83** | **0.83** | 0.93 | 0.92 | **0.92** | **0.92** | **0.93** | **0.93** | **0.93** | 0.98 | **0.98** | **0.98** | **0.98** | **0.98** | **0.98** | **0.98** |

***Table sI****: Power values for the three bioequivalence methods. namely ABE, PBE, and BBE method, with respect to the total variability (each columns) and the between batch variability, expressed as a percentage of the total variability (each row), for 3, 5, 7, and 10 batches. Bold values stand for BBE power greater or equals to ABE power and underscored values for BBE power greater or equal to PBE power.*

|  | ***3 batches*** | | | | | | | ***5 batches*** | | | | | | | ***7 batches*** | | | | | | | ***10 batches*** | | | | | | |
| --- | --- | --- | --- | --- | --- | --- | --- | --- | --- | --- | --- | --- | --- | --- | --- | --- | --- | --- | --- | --- | --- | --- | --- | --- | --- | --- | --- | --- |
| *Total var*  *BB var* | *5%* | *10%* | *15%* | *20%* | *25%* | *30%* | *35%* | *5%* | *10%* | *15%* | *20%* | *25%* | *30%* | *35%* | *5%* | *10%* | *15%* | *20%* | *25%* | *30%* | *35%* | *5%* | *10%* | *15%* | *20%* | *25%* | *30%* | *35%* |
| ***10%*** | 5.2 | 5.3 | 5.5 | 5.4 | 5.3 | 5.2 | 5.6 | 5.2 | 4.5 | 4.7 | 4.7 | 4.7 | 5.2 | 4.9 | 4.4 | 4.4 | 4.5 | 4.4 | 4.3 | 4.4 | 4.2 | 4.0 | 4.1 | 4.2 | 3.9 | 3.8 | 4.1 | 4.2 |
| ***20%*** | 5.3 | 5.5 | 5.3 | 5.6 | 5.6 | 5.5 | 5.2 | 4.7 | 4.7 | 4.8 | 5.1 | 4.8 | 5.0 | 5.1 | 4.3 | 4.2 | 4.8 | 4.5 | 4.4 | 4.6 | 4.2 | 3.9 | 3.8 | 4.0 | 4.0 | 4.2 | 4.0 | 4.3 |
| ***30%*** | 5.5 | 5.2 | 5.4 | 5.4 | 5.0 | 5.3 | 5.7 | 4.9 | 4.8 | 4.5 | 4.8 | 4.6 | 4.9 | 5.2 | 4.6 | 4.3 | 4.0 | 4.2 | 4.2 | 4.2 | 4.5 | 4.2 | 4.1 | 4.2 | 4.0 | 4.4 | 4.2 | 4.2 |
| ***40%*** | 5.7 | 5.3 | 5.4 | 5.5 | 5.6 | 5.7 | 5.7 | 4.6 | 4.5 | 4.6 | 5.0 | 4.8 | 5.1 | 5.0 | 4.1 | 4.3 | 4.7 | 4.0 | 4.8 | 4.2 | 4.4 | 4.3 | 4.2 | 4.2 | 4.4 | 3.7 | 4.0 | 4.3 |
| ***50%*** | 5.5 | 5.1 | 5.6 | 5.7 | 5.4 | 5.5 | 6.1 | 4.5 | 4.8 | 4.8 | 4.8 | 4.8 | 4.7 | 4.9 | 4.3 | 4.7 | 4.6 | 4.4 | 4.6 | 4.2 | 4.1 | 4.0 | 4.1 | 4.2 | 4.2 | 4.5 | 4.1 | 4.0 |
| ***60%*** | 5.2 | 5.2 | 5.7 | 5.0 | 5.1 | 5.4 | 5.8 | 4.6 | 4.8 | 5.1 | 5.1 | 4.8 | 4.9 | 5.0 | 4.9 | 4.6 | 4.6 | 4.5 | 4.4 | 4.3 | 4.3 | 4.1 | 4.0 | 4.0 | 3.9 | 4.2 | 4.0 | 3.7 |
| ***70%*** | 5.3 | 5.1 | 5.2 | 5.4 | 5.2 | 5.2 | 5.3 | 4.5 | 4.4 | 4.2 | 4.8 | 4.8 | 4.8 | 5.2 | 4.4 | 4.2 | 4.4 | 4.5 | 4.5 | 4.3 | 4.2 | 4.0 | 3.9 | 4.0 | 4.1 | 3.8 | 3.8 | 4.0 |
| ***80%*** | 5.2 | 5.5 | 5.5 | 5.4 | 5.4 | 5.6 | 5.7 | 4.8 | 4.6 | 4.9 | 5.1 | 5.0 | 4.8 | 4.6 | 4.7 | 4.2 | 4.6 | 4.5 | 4.4 | 4.3 | 4.4 | 4.1 | 4.0 | 4.5 | 4.0 | 3.8 | 3.9 | 4.1 |
| ***90%*** | 5.5 | 5.8 | 5.3 | 4.9 | 5.8 | 5.2 | 5.2 | 4.7 | 4.8 | 4.7 | 4.6 | 4.8 | 4.8 | 4.9 | 4.6 | 3.9 | 4.8 | 4.1 | 4.2 | 4.2 | 4.2 | 4.3 | 4.1 | 4.0 | 4.5 | 4.2 | 3.8 | 3.7 |

***Table sII****: Type I error values (%) for the Between-batch Bioequivalence method (BBE) with respect to the total variability (each columns) and the between batch variability, expressed as a percentage of the total variability (each row), for 3, 5, 7, and 10 batches.*

## Estimation of the goodness of mean estimation with sub-sample

The aim of this part is to validate the goodness of mean estimation using 6 samples in each batch instead of 10 samples. For this purpose, we used the Flonase data, where each batch is composed of 10 devices.

Left part of the figure

The following procedure was applied.

- Estimation of the mean of each batch with all the 10 units.
- Estimation the mean of each possible subset of 6 units for each batch, which induce 210 combinations.
- Computation of the ratio between these two means $\frac{\bar{x}_{6,i}}{\bar{x}_{10}}$.

We then obtain 23*210 ratios between the 6 units mean and the 10 devices mean.

Right part of the figure

The following procedure was applied.

- Estimation of the mean of each batch with all the 10 units.
- Estimation the mean of each possible subset of 6 units for each batch, which induce 210 combinations.
- Calculation of the Z-score for each mean calculated with 6 units as follow

$$\text{Z-score}\left( \bar{x}_{6,i} \right)=\frac{\left| \bar{x}_{6,i}-\bar{x}_{10} \right|}{s_{\bar{x}_{10}}},$$

where $i$ is the index for the combination.

- Computation of the mean of the Z-score per batch.

We then obtain 23 mean Z-score.

Results for Area (SP) are shown on the following figure.


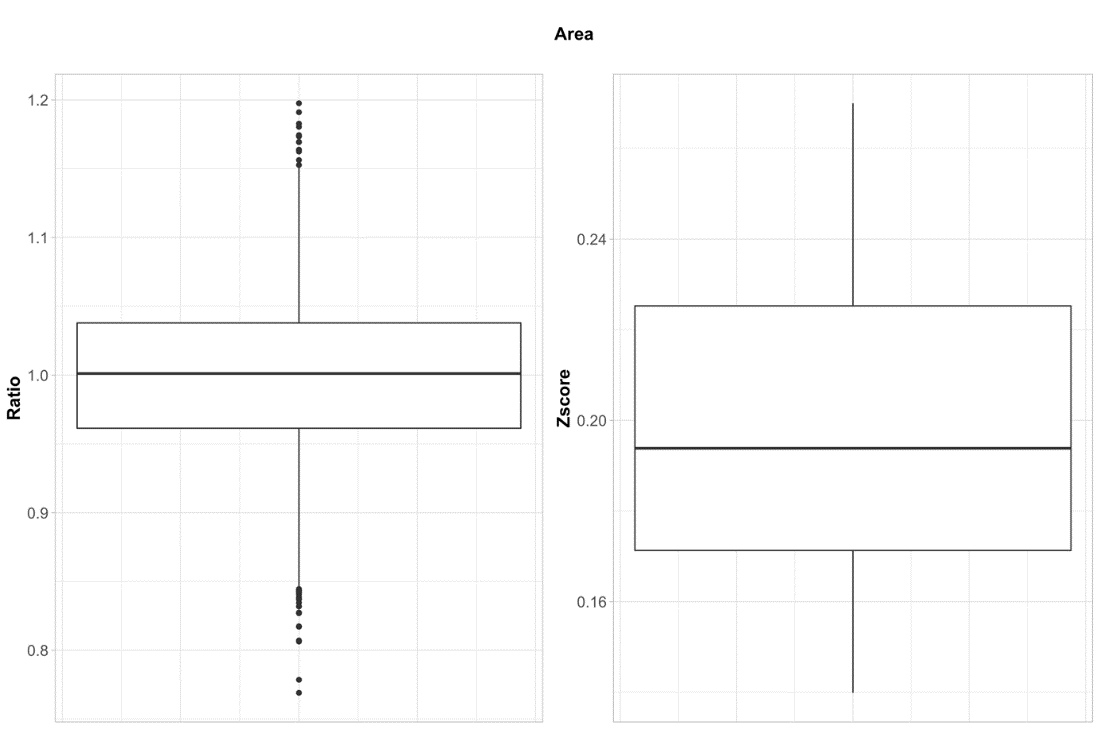


These results show that the mean estimated with 6 units remains close to the mean estimated using the available 10 units.

- Quite all the means are comprised between [85%, 115%] of the original mean.
- 50% of the means are comprised between [96.14%, 103.8%] of the original mean.

Furthermore, the Z-score study revealed that the difference between means with 6 units and means with 10 units is always lower than 27% of the between-batch variability (Z-score < 0.27).

Results for D50 (DSD) are shown on the following figure.


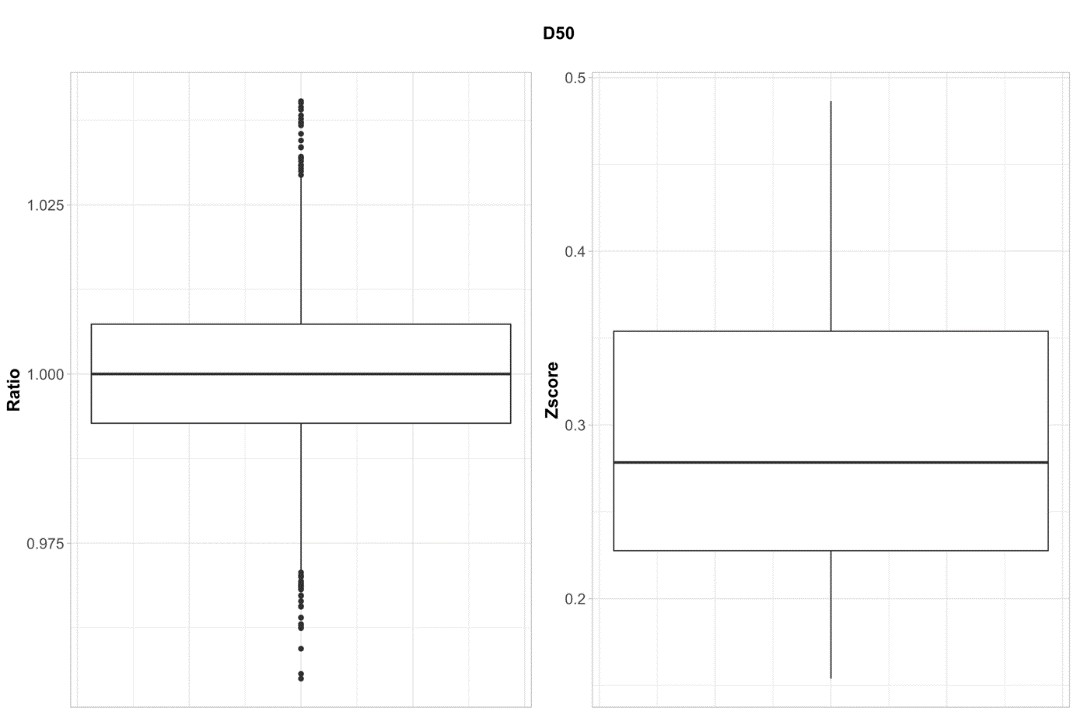


These results show that the mean estimated with 6 units remains close to the mean estimated using the available 10 units.

- Quite all the means are comprised between [97%, 103%] of the original mean.
- 50% of the means are comprised between [99.3%, 100.7%] of the original mean.

Furthermore, the Z-score study revealed that the difference between means with 6 units and means with 10 units is always lower than 49% of the between-batch variability (Z-score < 0.5).

In conclusion, this study proves that the estimates of the means are sufficiently accurate with 6 units per batch in comparison to 10 units per batch. At a constant sample size per product ($n_{R}=n_{T}= 30$), it seems more appropriate to take 5 batches of 6 units rather than 3 batches of 10 units.
